# Monocyte–macrophage dynamics as key in disparate lung and peripheral immune responses in severe anti‐melanoma differentiation‐associated gene 5‐positive dermatomyositis‐related interstitial lung disease

**DOI:** 10.1002/ctm2.70226

**Published:** 2025-02-04

**Authors:** Jia Shi, Xiaoya Pei, Jinmin Peng, Chanyuan Wu, Yulin Lv, Xiaoman Wang, Yangzhong Zhou, Xueting Yuan, Xingbei Dong, Shuang Zhou, Dong Xu, Jiuliang Zhao, Jun Liu, Jiao Huang, Bin Du, Chen Yao, Xiaofeng Zeng, Mengtao Li, Houzao Chen, Qian Wang

**Affiliations:** ^1^ Department of Rheumatology and Clinical Immunology Peking Union Medical College Hospital Chinese Academy of Medical Sciences & Peking Union Medical College National Clinical Research Center for Dermatologic and Immunologic Diseases (NCRC‐DID), Ministry of Science & Technology State Key Laboratory of Complex Severe and Rare Diseases Key Laboratory of Rheumatology and Clinical Immunology Ministry of Education Beijing China; ^2^ Department of Biochemistry and Molecular Biology State Key Laboratory of Medical Molecular Biology Institute of Basic Medical Sciences Chinese Academy of Medical Sciences and Peking Union Medical College Beijing China; ^3^ Medical Intensive Care Unit State Key Laboratory of Complex Severe and Rare Diseases Peking Union Medical College Hospital, Peking Union Medical College and Chinese Academy of Medical Sciences Beijing China; ^4^ State Key Laboratory of Protein and Plant Gene Research School of Life Sciences, Peking‐Tsinghua Center for Life Sciences Peking University Beijing China; ^5^ Department of Rheumatology Affiliated Hangzhou First People's Hospital Westlake University School of Medicine Hangzhou China; ^6^ College of Pulmonary and Critical Care Medicine Chinese PLA General Hospital Beijing China; ^7^ Department of Rheumatology and Clinical Immunology Peking Union Medical College Hospital Chinese Academy of Medical Sciences & Peking Union Medical College National Clinical Research Center for Dermatologic and Immunologic Diseases (NCRC‐DID) Ministry of Science & Technology State Key Laboratory of Common Mechanism Research for Major Diseases Key Laboratory of Rheumatology and Clinical Immunology Ministry of Education Beijing China

**Keywords:** anti‐MDA5 Antibody, dermatomyositis, macrophage, monocyte, single‐cell analysis

## Abstract

**Background:**

Anti‐melanoma differentiation‐associated gene 5‐positive dermatomyositis (anti‐MDA5+ DM) is a rare inflammatory autoimmune disorder often complicated by life‐threatening rapidly progressive interstitial lung disease (RP‐ILD). The underlying mechanisms driving immune dysfunction and lung injury, however, remain poorly understood. The study aims to gain insights into the disrupted immune landscape in peripheral and pulmonary compartments of severe anti‐MDA5+ DM and explore potential therapeutic targets.

**Methods:**

We employed single‐cell RNA sequencing to examine cellular constituents within five patients’ bronchoalveolar lavage fluid and paired peripheral blood mononuclear cells. Luminex assay and flow cytometry were further applied to validate the results.

**Results:**

Our analysis revealed starkly contrasting immune landscapes between the periphery and lungs, with peripheral immune suppression juxtaposed against pulmonary immune hyperactivation. Central to this dysregulation was the monocyte–macrophage lineage. Circulating monocytes exhibited an immunosuppressive phenotype, characterised by diminished cytokine production, reduced MHC II expression, and features resembling myeloid‐derived suppressor cells. These monocytes were recruited to the lungs, where they differentiated into monocyte‐derived alveolar macrophages (Mo‐AMs) with robust proinflammatory and profibrotic activities. Mo‐AMs drove cytokine storms and produced chemokines that amplified inflammatory cell recruitment and lung tissue remodelling. Additionally, peripheral T and NK cells exhibited increased cell death and active migration into the lungs, which may be the cause of lymphopenia.

**Conclusions:**

Our study underscores the pivotal role of monocyte–macrophage dynamics in the immunopathogenesis of anti‐MDA5+‐associated RP‐ILD, offering critical insights into compartment‐specific immune dysregulation. These findings suggest potential therapeutic strategies targeting monocyte recruitment and macrophage activation to mitigate disease progression.

**Key points:**

- Peripheral immune suppression and pulmonary immune hyperactivation characterise the distinct immune landscapes in anti‐MDA5+DM with RP‐ILD.
- Circulating monocytes transition from an immunosuppressive phenotype in the periphery to proinflammatory and profibrotic Mo‐AMs in the lungs.
- Chemokines produced by Mo‐AMs drive monocyte and other immune cell recruitment to the lungs, amplifying pulmonary inflammation.

## Graphical abstract

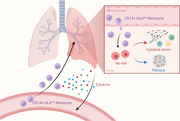

## 1 INTRODUCTION

Anti‐melanoma differentiation‐associated gene 5 antibody‐positive dermatomyositis, herein referred to as anti‐MDA5+ DM, represents a rare and distinctive subtype within the spectrum of idiopathic inflammatory myopathies (IIMs). Distinguished by its unique clinical profile, anti‐MDA5+ DM is notably characterised by minimal or absent muscular involvement and a profoundly perilous complication: rapidly progressive interstitial lung disease (RP‐ILD).^1^, ^2^ The scarcity of available human lung specimens, compounded by the paucity of animal models, has considerably constrained the scope of investigations into the underlying pathogenic mechanisms governing the development of RP‐ILD in anti‐MDA5+ DM, thereby underscoring the critical urgency and significance of advancing research in this domain.

MDA5, encoded by the IFIH1 gene, serves as a vital cytosolic sensor for viral RNA, orchestrating innate immune responses involving cytokine production, macrophage activation and helper T cell stimulation.^3^ In Coronavirus Disease 2019 (COVID‐19), MDA5 is one of the major sensors recognising SARS‐CoV‐2 infection and mediates interferon (IFN) response.^4^ COVID‐19 patients exhibit heightened innate immune responses in the respiratory tract.^5^ There are prominent similarities in clinical manifestations between anit‐MDA5+ DM and severe COVID‐19, such as lung pathogenic features and blood cytokine profiles.^6^ Moreover, the anti‐MDA5 antibody is also prevalent in COVID‐19 patients, with high titres associated with poor prognosis.^7^ While the precise pathogenic role of anti‐MDA5 antibodies in DM remains elusive, the associated ILD is characterised by elevated circulating ferritin levels, indicative of macrophage activation.^8^, ^9^ Besides, soluble factors from activated macrophages, such as neopterin, chitotriosidase, CD163 and CD206, have been linked to disease activity and poor prognosis.^10^, ^11^, ^12^, ^13^ Anti‐MDA5+ DM shares features with macrophage activating syndrome (MAS), such as lymphopenia and elevated circulating cytokines (IL1, IL6, IL8, IL18 and IFN‐α),^14^ albeit not meeting all the diagnostic criteria of MAS.^15^

Recently, Zuo et al.^16^ conducted immunohistochemical analyses of the lung tissue from anti‐MDA5+ DM patients with ILD, providing direct evidence of alveolar macrophage activation and CD163‐positive macrophage accumulation. Gono et al.^17^ identified a self‐sustaining proinflammatory network driven by activated monocytes/macrophages through integrated miRNA‐mRNA association analysis. Most notably, Ye et al. conducted single‐cell resolution studies, focusing on peripheral T and B cells. Their finding unveiled a distinctive adaptive immune landscape characterised by autoantigen‐driven antibody responses, heightened type I IFN signalling and aberrant metabolic remodelling.^18^ These combined efforts deepen our understanding of the immunopathogenesis of anti‐MDA5+ DM, highlighting the complex interplay between innate and adaptive immune mechanisms.

In this context, several pivotal research challenges warrant attention. First, it is crucial to distinguish between peripheral and pulmonary immune dysregulation, as peripheral abnormalities often represent consequences rather than primary pathogenic factors. Second, the research on COVID‐19 pathology highlights the importance of investigating immune cell profiles within bronchoalveolar lavage fluid (BALF), particularly to gain deeper insights into intrapulmonary pathology. Remarkably, the lack of BALF‐focused research in the context of anti‐MDA5+ DM points to a significant research gap.

In this study, we aimed to elucidate the altered immune landscape in both peripheral and pulmonary domains in individuals with severe anti‐MDA5+ DM. We used single‐cell RNA sequencing to analyse cellular constituents in patients’ BALF and paired peripheral blood mononuclear cells (PBMCs). By integrating data from these two sites, our primary focus was elucidating potential regulatory pathways and interaction networks. Our study yielded pivotal insights into the perturbed monocyte–macrophage populations associated with this condition.

## 2 RESULTS

### 2.1 Overview of immune cell landscapes in peripheral blood and lung

**FIGURE 1:**
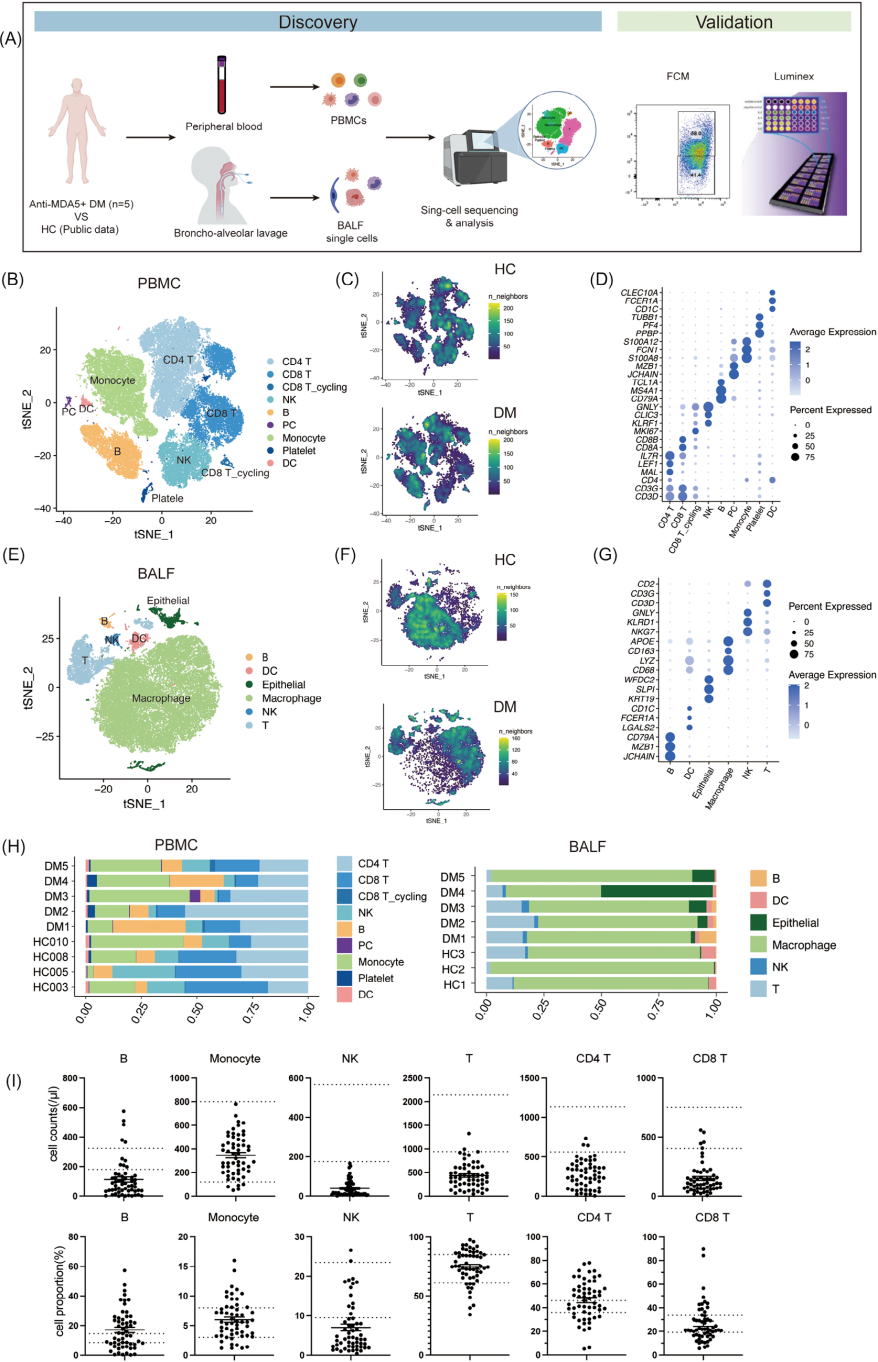
Overview of immune cell landscapes in peripheral blood and lungs. (A) Schematic representation of the study design, including sample collection, single‐cell transcriptomic analysis and validation experiments. (B and E) t‐SNE plots visualising cell clusters from integrated single‐cell transcriptomes of PBMCs (B) and BALF (E) obtained from anti‐MDA5+ DM patients and healthy controls (HCs). (C and F) t‐SNE plots showing the single‐cell transcriptomes of PBMCs (C) and BALF (F) from HCs (top) and anti‐MDA5+ DM patients (bottom). (D and G) Dot plots showing key marker genes used to define each immune cell type in PBMCs (D) and BALF (G), corresponding to the clusters in panels B and E. (H) Bar charts showing the proportions of immune cell clusters in PBMCs (left) and BALF (right) from anti‐MDA5+ DM patients and HCs. (I) Immune cell counts (top row) and proportions (bottom row) in peripheral blood from 57 anti‐MDA5+ DM inpatients, compared with normal ranges (indicated by dashed lines).

To characterise the nature of circulating and pulmonary immune cell populations in anti‐MDA5+ DM patients with RP‐ILD, we performed scRNA‐seq (10X Genomics) to analyse paired PBMCs and BALF from five patients. Patients’ clinical data were recorded and presented in Table S1. A high‐quality scRNA‐seq dataset, comprising 82 819 cells (44 689 cells from PBMCs and 38 130 cells from BALF), enabled us to delineate the immune landscape in both peripheral and lung domains (Figure 1A). We contrasted these profiles with datasets from healthy controls (HCs), particularly with GSE158055 for PBMCs (HC003, HC005, HC008 and HC010) and GSE145926 for BALF (HC1, HC2 and HC3). The demographic information of HCs is provided in Table S2.

In PBMCs, nine major cell types, namely CD4 T cells, CD8 T cells, CD8 T_cycling cells, natural killer (NK) cells, monocytes, B cells, platelets, plasma cells (PCs) and dendritic cells (DCs), were discerned and annotated through established marker genes (Figures 1B,C,D and S1A). Likewise, in BALF, six predominant cell types comprising macrophages, T cells, NK cells, DCs, epithelial cells and B cells were identified and characterised using the same methodology (Figures 1E,F,G and S1C).

Significant differences were evident between HCs and anti‐MDA5+ DM patients based on t‐distributed stochastic neighbour embedding (t‐SNE) projections (Figures 1C,F and S1B,D). In PBMC, the most prominent change was a reduction of NK cell populations, although the difference in NK cell ratio did not reach statistical significance (Figures 1H and S1E). Further analysis of clinical data from 57 additional anti‐MDA5+ DM inpatients corroborated these findings, revealing a general decrease in NK and T cell counts, including CD4 T and CD8 T cells (Figure 1I), aligning with previous studies.^9^, ^19^ On the contrary, in BALF, we found enrichment of NK cells in patients, especially in DM1, DM2 and DM3, diverging from the HCs’ profiles (Figures 1H and S1F). Notably, despite macrophages being the most populous in BALF for both groups, their transcriptional profiles varied significantly (Figure 1F). Together, these data reveal great changes in periphery blood and lung immune cell compartments compared with HCs.

### 2.2 Regional pulmonary hyperinflammation in contrast to peripheral blood mononuclear cells

**FIGURE 2:**
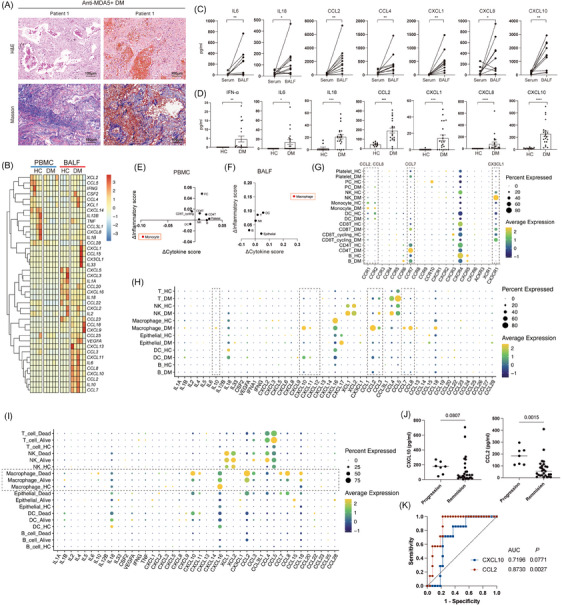
Regional pulmonary hyperinflammation in contrast to PBMCs. (A) Representative histologic images of lung samples from patients with anti‐MDA5+ DM, stained with haematoxylin and eosin (top) and Masson's trichrome (bottom). Scale bar: 100 µm. (B) Heatmap showing the expression levels of key cytokines and chemokines associated with cytokine storm in PBMCs and BALF from HCs and patients. Row‐wise (gene‐wise) scaling was applied to enhance visual contrast within each cytokine. (C) Scatter plots comparing cytokine and chemokine levels in paired BALF and serum samples from anti‐MDA5+ DM patients (*n* = 10). Values below the detection limit are assigned as 0. (D) Scatter plots showing the levels of selected cytokines and chemokines in serum samples from anti‐MDA5+ DM patients (*n* = 20) and HCs (*n* = 10). Values below the detection limit are assigned as 0. (E & F) ΔInflammatory and Δcytokine scores for cell clusters in PBMCs (E) and BALF (F), calculated as the difference between patients and HCs. The zero axis represents the HC baseline, with greater deviations indicating larger differences. (G) Dot plot of chemokine receptor gene expression in PBMC cell clusters from anti‐MDA5+ DM patients and HCs, based on the scRNA‐seq data. Some ligand–receptor pairs are indicated by dashed boxes. The bubble size represents the percentage of cells expressing each gene within a cluster, while the colour intensity indicates the average expression level. (H) Dot plot showing the expression of indicated cytokines and chemokines in BALF cell clusters from anti‐MDA5+ DM patients and HCs based on scRNA‐seq data. (I) Expression of indicated cytokines and chemokines across BALF cell clusters from alive patients, deceased patients and HCs based on scRNA‐seq data. (J) Scatter plots showing serum levels of CXCL10 (left) and CCL2 (right) in anti‐MDA5+ DM patients with disease progression versus remission. (K) ROC curves illustrating the performance of CXCL10 and CCL2 for distinguishing disease progression from remission, with corresponding AUC values and *p* values.

Anti‐MDA5+ DM‐associated RP‐ILD could progress to respiratory failure, which is featured by cytokine storm syndrome.^20^ Histopathological analysis of lung biopsy samples from these patients demonstrated diffuse alveolar damage, fibroproliferative responses and the presence of fibrotic foci (Figure 2A). High‐resolution computed tomography (HRCT) further corroborated these findings, showing characteristic patterns of diffuse ground‐glass opacities and consolidations (Figure S2A).

We compared gene expressions of cytokine and chemokine in samples from HCs and patients (Figure 2B). The scRNA‐seq data from BALF indicated elevated levels of interleukins (*IL6, IL10*) and *TNF*, alongside a range of chemokines (*CCL2, CCL3, CCL4, CCL7, CCL8, CXCL10* and *CXCL11*) in patients compared with HCs. This pattern was distinct from that in PBMCs, suggesting a hyperinflammatory state localised to the lung. Luminex assay measurements of BALF and serum in patients aligned with the scRNA‐seq data, showing higher concentrations of inflammatory cytokines and chemokines (IL6, IL18, CCL2, CCL4, CXCL1, CXCL8 and CXCL10) in BALF than in paired serum (Figures 2C and S2B). Compared with HCs, the concentrations of inflammatory cytokines and chemokines in patients’ serum were elevated, particularly for IFN‐α, IL6, IL18, CCL2, CXCL1, CXCL8 and CXCL10 (Figures 2D and S2C). However, an analysis of the transcriptome data from PBMCs of DM patients did not reveal a significant inflammatory state compared with HCs (Figure 2B). This suggests that the proinflammatory cytokines detected in the serum are likely derived from lung tissue cells rather than from peripheral blood cells.

We next aimed to identify the cellular sources of cytokine production in the patients’ lungs. Cytokine and inflammatory scores were calculated for each cell cluster based on cytokine expression and inflammatory response genes, respectively, to assess their contribution to the inflammatory cytokine storm^21^ (Figures 2E,F). Our analysis revealed significantly elevated expression of these cytokine and inflammatory genes among various immune cells and epithelial cells in patients’ BALF, signifying an intense inflammatory response in the lung (Figure 2F). Notably, macrophages in patients’ BALF samples exhibited substantially higher cytokine and inflammatory scores, indicating their pivotal role in driving inflammatory storm. In contrast, peripheral monocytes demonstrated markedly lower cytokine and inflammatory scores than those in HCs, reflecting their functional impairments and a state of cytokine deficit (Figures 2E and S2D). This disparity underscores the localised nature of the hyperinflammatory response in the lung, which is distinct from the peripheral immune landscape observed in PBMCs.

Given the significant increase in multiple chemokine concentrations in BALF, we assessed gene expression levels of chemokine receptors of PBMC (Figure 2G). The receptor *CCR7* of T cells and *CX3CR1* of NK cells were substantially upregulated in anti‐MDA5+ DM patients, suggesting that the observed reduction in circulating T and NK cells might be attributed to their migration to the lung. Furthermore, monocytes demonstrated elevated expression of *CCR1* and *CCR2*, supporting the hypothesis of monocyte recruitment to the pulmonary microenvironment. Besides, our examination of chemokine expressions across different cell clusters in BALF revealed that lung macrophages in patients are likely key players in local inflammation (Figure 2H). They exhibit high transcriptional levels of chemokines such as *CXCL10*, *CXCL11*, *CCL2*, *CCL3*, *CCL7* and *CCL8*, which may facilitate the recruitment of additional monocytes and T cells to the lungs, thereby amplifying the inflammatory response.

To identify potential prognostic markers, we analysed differences in cytokine and chemokine expression levels in BALF between surviving and deceased patients. Transcriptomic analysis indicated significantly elevated expression of *CXCL10* and *CCL2* in BALF macrophages from deceased patients compared with survivors (Figure 2I). Based on the hypothesis mentioned above, the cytokine expression in BALF could be indirectly reflected by serum levels, and since peripheral blood samples are easier to obtain, we used serum cytokine levels to validate the findings. Patients were then further stratified into progression and remission groups based on follow‐up pulmonary function data. Although the sample size was limited, CCL2 levels were notably higher in the progression group compared with the remission group (Figure 2J). Receiver operating characteristic (ROC) curve analysis demonstrated that CCL2 effectively distinguished patients with disease progression from those in remission, achieving an area under the curve (AUC) of 0.873 (Figure 2K). Furthermore, univariate logistic regression analysis identified CCL2 as a significant predictor of ILD progression (odds ratio [OR] = 1.012, 95% confidence interval [CI]: 1.003–1.025, *p *= .0247). In contrast, CXCL10 did not exhibit significant predictive value, potentially due to the small sample size (OR = 1.001, 95% CI: 0.996–1.006, *p *= .5544).

These findings highlight a discrepancy between peripheral immune dysregulation and severe lung inflammation in anti‐MDA5+ DM patients, emphasising the localised nature of the pulmonary response. Peripheral blood serves more as an indirect indicator than a primary disease driver. These results emphasise the importance of focusing on BALF analyses to better understand the mechanisms of anti‐MDA5+ ILD.

### 2.3 Pulmonary monocyte–macrophage lineage mediates inflammation and fibrosis

**FIGURE 3:**
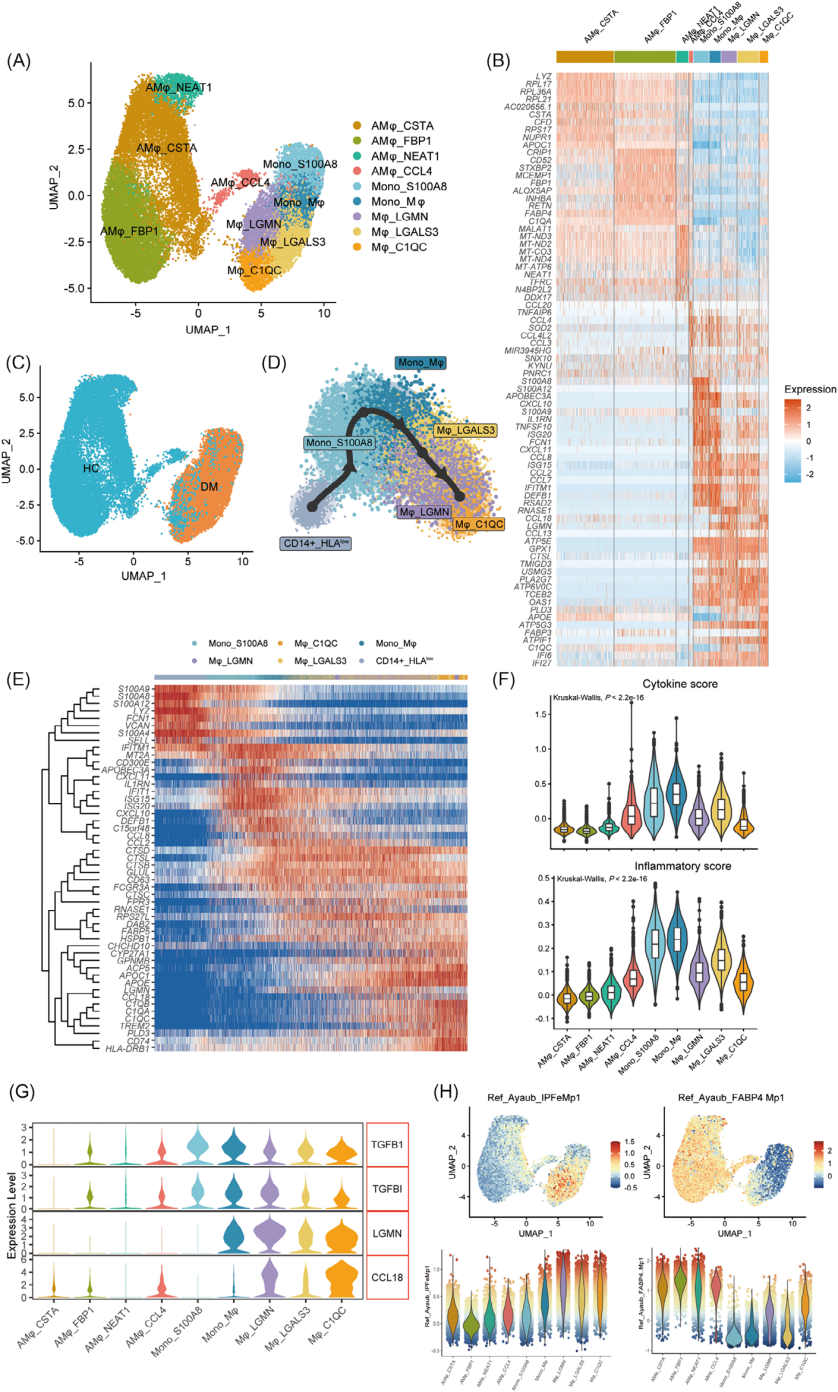
Pulmonary monocyte–macrophage lineage mediates inflammation and fibrosis. (A) UMAP plot illustrating nine monocyte–macrophage cell clusters in BALF samples. (B) Heatmap showing the top 10 marker genes for each monocyte–macrophage cluster. (C) UMAP plot showing the distribution of monocyte–macrophages in BALF samples from HCs and patients. (D) Pseudo‐time trajectory analysis of monocyte–macrophages in anti‐MDA5+ DM patients. (E) Heatmap of differentially expressed genes along the pseudo‐time trajectory, annotated with pseudo‐time progression and cell type. (F) Violin plots comparing cytokine scores (top) and inflammatory scores (bottom) across monocyte–macrophage clusters. (G) Violin plots showing the expression levels of fibrosis‐related genes (*TGFB1, TGFBI, LGMN* and *CCL18*) across monocyte–macrophage clusters. (H) Gene set module scores for “IPF‐expanded macrophages” (IPFe‐Mp, left) and alveolar FABP4+ macrophages (FABP4 Mp, right)^23^ visualised on UMAP embeddings (top) and as violin plots (bottom) across monocyte–macrophage clusters. Dot colour reflects module score intensity, while violin colours represent cluster identity.

Given the pivotal role of macrophages in the cytokine storm, we conducted a more detailed analysis of monocyte–macrophages (Mφ) lineages in BALF, identifying nine distinct populations with unique gene expression profiles (Figures 3A,B and S3A). The Monocyte_S100A8 (Mono_S100A8) population showed high levels of *CD14*, alarmins (*S100A8, S100A9, S100A12*), IFN‐stimulated genes (ISGs) (*ISG20, ISG15, IFITM1*) and proinflammatory cytokines (*CXCL10, CXCL11, CCL2, CCL7*), indicating a robust inflammatory response. The neighbouring Mono_Mφ population exhibited a less distinct phenotype, indicating a transitional state of differentiation. Furthermore, we identified three subsets of monocyte‐derived alveolar macrophages (Mo‐AM): Mφ_LGMN, Mφ_LGALS3 and Mφ_C1QC, primarily expressing *CD68* and ISGs (*IFI6, IFI20*). Additionally, four types of alveolar macrophages (AMφ), AMφ_CCL4, AMφ_NEAT1, AMφ_FBP1 and AMφ_CSTA, were characterised, notably expressing *FABP4* and *MARCO*. In patients, Mono_S100A8, Mono‐Mφ and Mo‐AMs were predominant, whereas, in HCs, the alveolar compartment was mainly occupied by AMφ (Figures 3C and S3B), highlighting a shift in macrophage populations in the disease state. The discrepancy of the DM5 sample in cell proportion compared with the other four DM BALF samples may be attributed to this sample capturing only a limited number of high‐quality cells.

In order to obtain the differentiation trajectory of circulating and BALF monocyte–macrophages, we used Slingshot for pseudo‐time analysis. This revealed that peripheral monocytes (CD14+_HLA^low^ monocytes, which will be detailed discussed in the next section) underwent a differentiation trajectory toward Mono_S100A8, Mφ_LGALS3, Mφ_LGMN and Mφ_C1QC in the BALF, aligning with the theory of peripheral monocytes recruitment to inflammatory tissues (Figures 3D and S3C). Differential gene expression analysis along the monocyte–macrophage trajectory revealed four key stages (Figure 3E). The initial stage was marked by heightened expression of calprotectin (*S100A4, S100A8, S100A9, S100A12*) and oxidative stress marker (*MT2A*), indicating an early inflammatory response. This was followed by upregulation of proinflammatory markers (*CCL2, CCL8, CXCL10, CXCL11, CD300E, SELL*), antimicrobial genes (*DEFB1, LYZ*) and ISGs (*IFIT1, IFITM1, ISG15, ISG20*), suggesting a hyperinflammatory state. In the third stage, an increase in cathepsins (*CTSD, CTSL, CTSB*) and stress‐response marker (*HSPB1*) was observed. Finally, in the later stage, macrophages expressed genes related to fibrosis (*CCL18, LGMN*), lipid metabolism (*APOE, APOC1, TREM2, FABP5, PLD3*), antigen processing and presentation (*CD74, LGMN, TREM2*) and complement activation (*C1QA, C1QB, C1QC, CD74*), signalling a shift towards tissue remodelling and immune modulation. To decipher the specific roles of each monocyte and macrophage cluster in severe lung inflammation, we calculated inflammatory and cytokine scores of each group (Figure 3F). Our analysis revealed that the Mono_S100A8, Mono_Mφ and Mo‐AM clusters exhibited higher scores than the AMφ, suggesting that these recruited monocytes may be the primary contributors to the cytokine storm in the lungs.

Beyond the acute inflammatory response, anti‐MDA5+ DM patients with ILD also suffered from accompanied pulmonary fibrosis, which is linked to worse prognoses.^22^ Assessing fibrosis‐related gene (*TGFB1, TGFBI, LGMN* and *CCL18*) expression of each cell cluster, we found elevated levels in Mo‐AMs (Mφ_LGMN, Mφ_LGALS3 and Mφ_C1QC) (Figure 3G), pointing to their potential role in fibrogenesis. To further delineate functional polarisation, we assessed the M1 and M2 phenotypic scores of the five clusters. Mono_S100A8 and Mono‐Mφ populations predominantly exhibited an M1 phenotype, while Mo‐AM clusters showed higher expression of M2 markers (Figure S3D). Notably, macrophages in fibrotic niches of pulmonary fibrosis exhibit shared transcriptional programs.^23^, ^24^, ^25^ To further investigate, we compared the transcriptional profiles of these Mo‐AM clusters with publicly available datasets of idiopathic pulmonary fibrosis (IPF). Using gene set expression‐based cell scores, we identified significant differences across populations (Figures 3H and S3E). This analysis indicated that Mφ_LGALS3, Mφ_LGMN and Mφ_C1QC populations closely resembled IPF‐specific macrophage phenotypes, whereas Mono_S100A8 and AMφ subsets were more similar to homeostatic monocytes and alveolar macrophages, respectively. This continuum of gene expression, transitioning from proinflammatory to profibrotic gene expression along the monocyte–macrophage trajectory, highlights the multifaceted role of these cells in disease progression and tissue repair.

To further assess the functional status of these cells, we analysed their enriched genes using the Gene Ontology Biological Process (GO‐BP) database (Figure S3F). This revealed functional heterogeneity and specialisations: Mono_S100A8 and Mono‐Mφ clusters were associated with cytokine production and antiviral responses, while Mφ_LGMN and Mφ_LGLAS3 were enriched in energy metabolism and protein/lipid regulation pathways, respectively. Mφ_C1QC was involved in antigen presentation and lipid metabolism.

Overall, these data highlight a notable accumulation of monocytes and Mo‐AMs in the lungs of anti‐MDA5+ DM patients with ILD. These cells appear central to innate immune activation, driving both inflammation and tissue fibrosis progression.

### 2.4 Peripheral expansion of myeloid‐derived suppressor cell‐like monocytes

**FIGURE 4:**
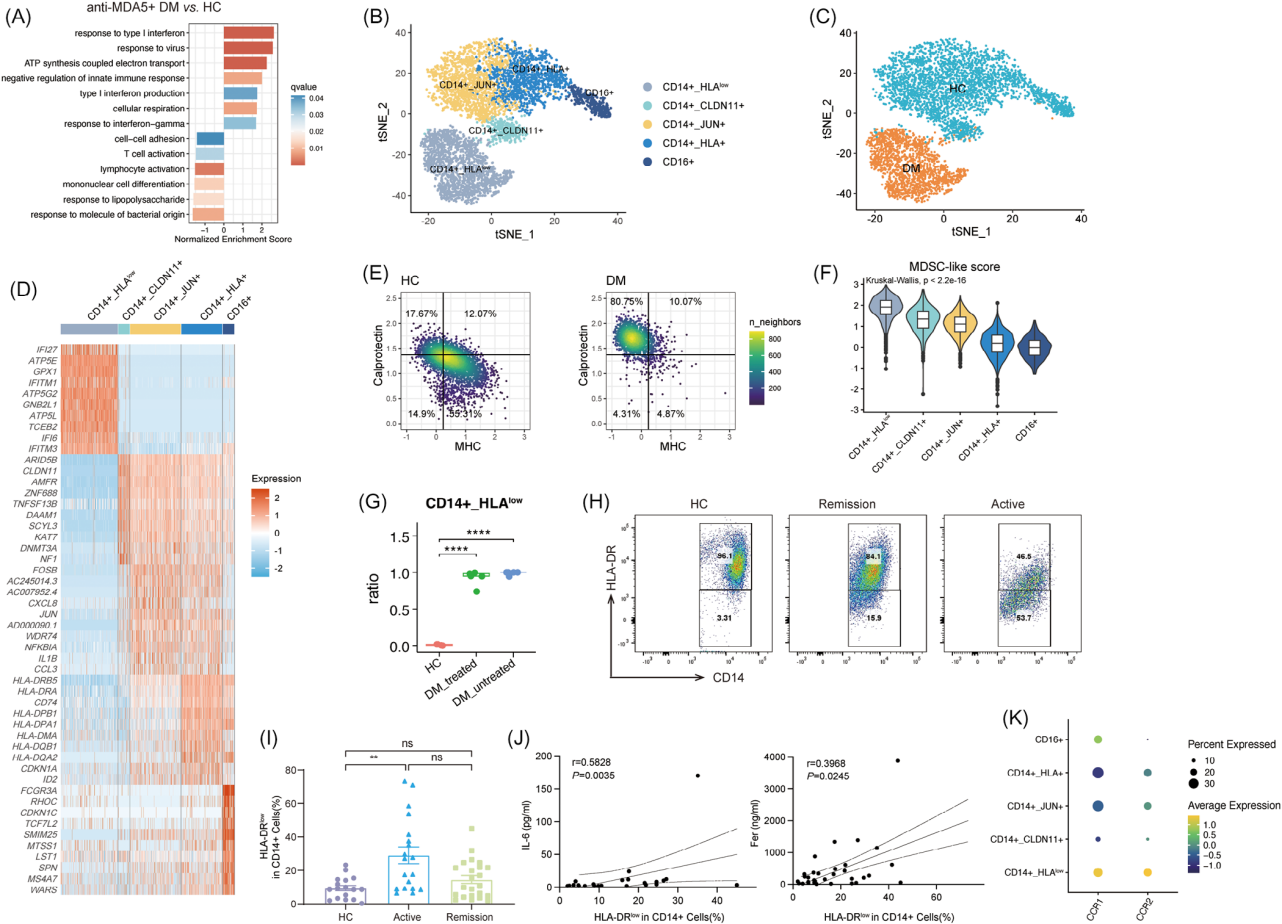
Peripheral expansion of MDSC‐like monocytes in anti‐MDA5+ DM. (A) Bar plot showing pathways enriched in monocytes from anti‐MDA5+ DM patients compared with HCs. (B) t‐SNE plot showing five distinct monocyte clusters identified in PBMCs. (C) t‐SNE plot illustrating the distribution of monocytes across HCs and anti‐MDA5+ DM patients. (D) Heatmap presenting the top 10 marker genes for each monocyte cluster. (E) Density plots of peripheral monocytes showing composite MHC II and calprotectin signature scores, mapped to two‐dimensional spaces. Median scores define the quadrants, and percentages of cells in each are indicated. (F) Violin plot demonstrating MDSC‐like scores of each monocyte cluster. (G) The proportion of CD14+_HLA^low^ monocytes among peripheral monocytes in treated patients, untreated patients and HCs. (H) Representative flow cytometry plot showing HLA‐DR expression in CD14+ PBMCs from HC, remission and active anti‐MDA5+ DM groups. (I) Comparison of HLA‐DR^low^ percentages in CD14+ PBMCs among anti‐MDA5+ DM‐active (*n* = 19), remission (*n* = 23) and HC (*n* = 17) groups. (J) Spearman correlation analysis of HLA‐DR^low^ percentages in CD14+ cells and serum levels of IL‐6 (left) and ferritin (right). (K) Dot plot showing *CCR1* and *CCR2* gene expression levels across the monocyte clusters, with dot size indicating the percentage of cells expressing the gene and colour representing average expression intensity.

We then delved into the molecular features of peripheral monocytes in anti‐MDA5+ DM patients. Our transcriptome analysis identified a distinct expression profile with 747 genes upregulated and 950 genes downregulated compared with controls. GO‐BP pathway analysis of the upregulated differentially expressed genes (DEGs) highlighted their involvement in the antiviral mechanism, supporting the hypothesis that viral infections may be a trigger for anti‐MDA5+ DM pathogenesis.^26^, ^27^ In addition, these monocytes showed enrichment in type I IFN and IFN‐γ signalling pathways, as well as energy metabolism pathways (Figure 4A). In contrast, the downregulated DEGs were associated with response to lipopolysaccharide and lymphocyte activation, implying possible functional impairments in these cells (Figure 4A). Further analysis through re‐clustering led to the identification of five distinct monocyte subtypes: CD14+_HLA^low^ monocyte, CD14+_CLDN11+ monocyte, CD14+_JUN+ monocyte, CD14+_HLA+ monocyte and CD16+ non‐classical monocyte (ncMon) (Figures 4B,D and S4A). The t‐SNE analysis showed perturbed transcriptome features in monocytes from anti‐MDA5+ DM patients compared with controls (Figure 4C). Specifically, monocytes from patients expressed higher levels of *PLAC8* and *MPO*, markers associated with immature states, alongside anti‐inflammation markers such as *CD163*, *SELL* and *PLBD1* ^28^ (Figure S4C).

The composition of monocyte clusters in anti‐MDA5+ DM patients differed significantly from that of HCs (Figures 4C and S4B). A distinctive lack of ncMon (Figure S4B) was noted in anti‐MDA5+ DM patients, which was previously linked to inflammation resolution.^29^ Flow cytometry results further confirmed a reduced presence of CD16+ ncMon in these patients (Figure S4D), consistent with the earlier study.^30^ Intriguingly, the percentage of CD16+ ncMon in monocytes was inversely correlated with IL‐6 levels, reflecting the disease activity and inflammation severity (Figure S4D).

The CD14+_HLA^low^ cluster, which formed the largest proportion of monocytes in anti‐MDA5+ DM patients, exhibited a unique profile with overexpression of canonical ISGs such as *IFI27, IFI6*, *IFITM1* and *IFITM3*, while showing reduced expression of cytokine/chemokine genes like *IL1β* and *CXCL8* (Figure 4D). Additionally, it was characterised by lower expression of MHC II molecules (*HLA‐DPA1*, *HLA‐DPB1* and *HLA‐DR*), a hallmark of myeloid‐derived suppressor cells (MDSCs) (Figure 4D). MDSCs are a heterogeneous group of immature myeloid cells that expand in response to inflammatory conditions, known for their immunosuppressive properties and capability to suppress T cell responses.^31^ The distinctive features of CD14+_HLA^low^ monocytes, including lower composite scores for MHC II molecules, higher scores for calprotectin (Figures 4E and S4E), and functional annotations revealed by GO enrichment analysis (Figure 4A), closely resembled those of MDSCs. This cluster also had the highest MDSC‐like score among all five clusters (Figure 4F).

Notably, the five patients included in our study had received immunosuppressive therapy for a relatively long duration, raising the question of whether the emergence of MDSC‐like monocytes is a consequence of the treatment or an intrinsic feature of anti‐MDA5+ DM‐ILD. To address this, we integrated scRNA‐seq data of PBMCs from five treatment‐naïve anti‐MDA5+ DM‐ILD patients with our own PBMC data and publicly available HCs. The detailed clinical data of these patients can be found in Table S3. Using our established clustering approach, we re‐analysed the monocyte populations (Figure S4F). The t‐SNE projection showed a substantial overlap in monocyte distributions between treated and untreated patients, suggesting shared features (Figure S4G). Moreover, four out of the five untreated patients exhibited nearly 100% CD14+_HLA^low^ monocytes, a proportion significantly higher than that observed in HCs (Figures 4G and S4H). These findings further confirm a fundamental shift in the functional state and subtype composition of peripheral monocytes in patients with anti‐MDA5+ DM‐ILD.

Flow cytometry further confirmed an increased percentage of HLA‐DR^low^ cells in CD14+ monocytes from anti‐MDA5+ DM patients (Figure 4H,I), correlating positively with serum ferritin and IL‐6 levels (Figure 4J). The relations between the percentage of HLA‐DR^low^ monocytes and other inflammatory indexes are shown in Figure S4I.

Given the elevated levels of monocyte‐attracting chemokines (e.g., CCL2) in BALF (Figure 2C) and upregulation of *CCR1* and *CCR2* in circulating monocytes (Figure 2G), we then analysed *CCR1* and *CCR2* expression across monocyte clusters to identify the cluster most likely to migrate to the lung. Among these, the CD14+_HLA^low^ cluster exhibited the highest expression of *CCR1* and *CCR2* (Figure 4K). Moreover, the Mono_S100A8 cluster, which represents recruited monocytes in the lung, closely resembles CD14+_HLA^low^ monocytes, characterised by low MHC II expression and elevated levels of calprotectin (*S100A8, S100A9, S100A12*) (Figure 3B). Taken together, these findings support the hypothesis that MDSC‐like monocytes are actively recruited to the lung, where they further differentiate into Mo‐AMs.

Overall, these findings indicate that the circulating monocytes in anti‐MDA5+ DM patients exhibit significant functional alterations, characterised by heightened ISG responses, reduced expression of cytokines and diminished MHC II molecule levels, suggesting a shift toward an immunosuppressive phenotype. Furthermore, these monocytes are actively recruited to the lungs, where they differentiate into Mo‐AMs, potentially contributing to local immune dysregulation and the pathogenesis of anti‐MDA5+ DM‐ILD.

### 2.5 Dysregulated cell death and active migration to the lung of peripheral NK and T cells

**FIGURE 5:**
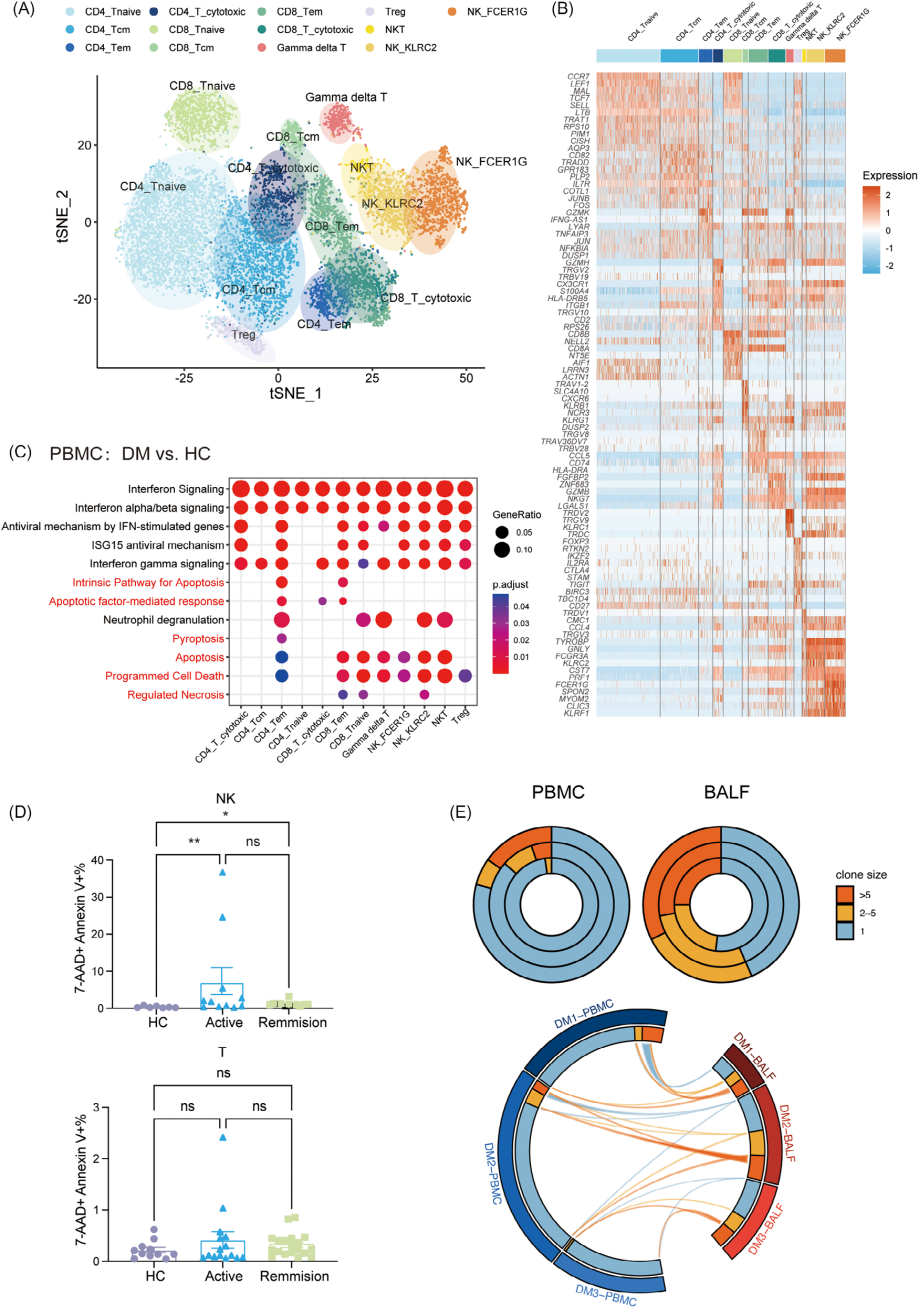
Dysregulated cell death and active migration of peripheral NK and T cells to the lung in anti‐MDA5+ DM. (A) t‐SNE plot showing T and NK cell clusters identified in PBMCs. (B) Heatmap showing the top 10 marker genes for each T and NK cell cluster. (C) Representative Gene Ontology (GO) pathways enriched by the highly expressed genes in T and NK subtypes. Dot size indicates gene ratio, and colour represents adjusted *p* values. (D) Flow cytometry analysis of 7‐AAD and annexin V staining in NK and T cells. (E) Donut plots (top) representing T cell clonal expansion in PBMCs and BALF from anti‐MDA5+ DM patients, with rings representing individual patients. The circus plot (bottom) displays TCR clonotype sharing between PBMCs and BALF.

We investigated the dynamics of NK and T lymphocytes, observing reduced absolute cell counts in anti‐MDA5+ DM patients (Figure 1I).^9^, ^19^ Our detailed clustering of those cells identified 13 subsets in PBMCs, revealing significant heterogeneity (Figures 5A,B and S5A). This diversity is evident in the varied ratios of each cell cluster (Figure S5B,C).

Further exploration into the T and NK lymphopenia (Figure 1I), involved comparing the functional phenotypes of these subsets, focusing on enriched pathways in upregulated genes. In addition to IFN signalling and immune response pathways, we also detected a marked increase in cell death pathways, including apoptosis, pyroptosis and necrosis (Figure 5C). Consistently, the percentage of late apoptotic NK cells was higher in anti‐MDA5+ DM patients of both active and remission states than in HCs, with the percentage of late apoptotic T cells being a little higher in patients though not reaching statistical significance (Figure 5D). This finding suggests an elevated incidence of cell death among NK and T cells in anti‐MDA5+ DM.

Utilising T‐cell receptor sequencing (TCR‐seq), we assessed the clonal expansion and clonotype sharing between PBMC and BALF samples in patients. Compared with the periphery, pulmonary T cells showed more pronounced clonal expansion and shared clonotypes with their paired peripheral blood counterparts (Figure 5E). This, combined with higher chemokine levels in BALF, suggests a likely recruitment of T cells from the peripheral to the lungs.

On the whole, these findings indicate that the reduction in circulating NK and T cells in anti‐MDA5+ DM patients may be attributed to both augmented cell death and active migration to the lungs.

### 2.6 B cell dynamics and abnormal autoantibody response in BALF

**FIGURE 6:**
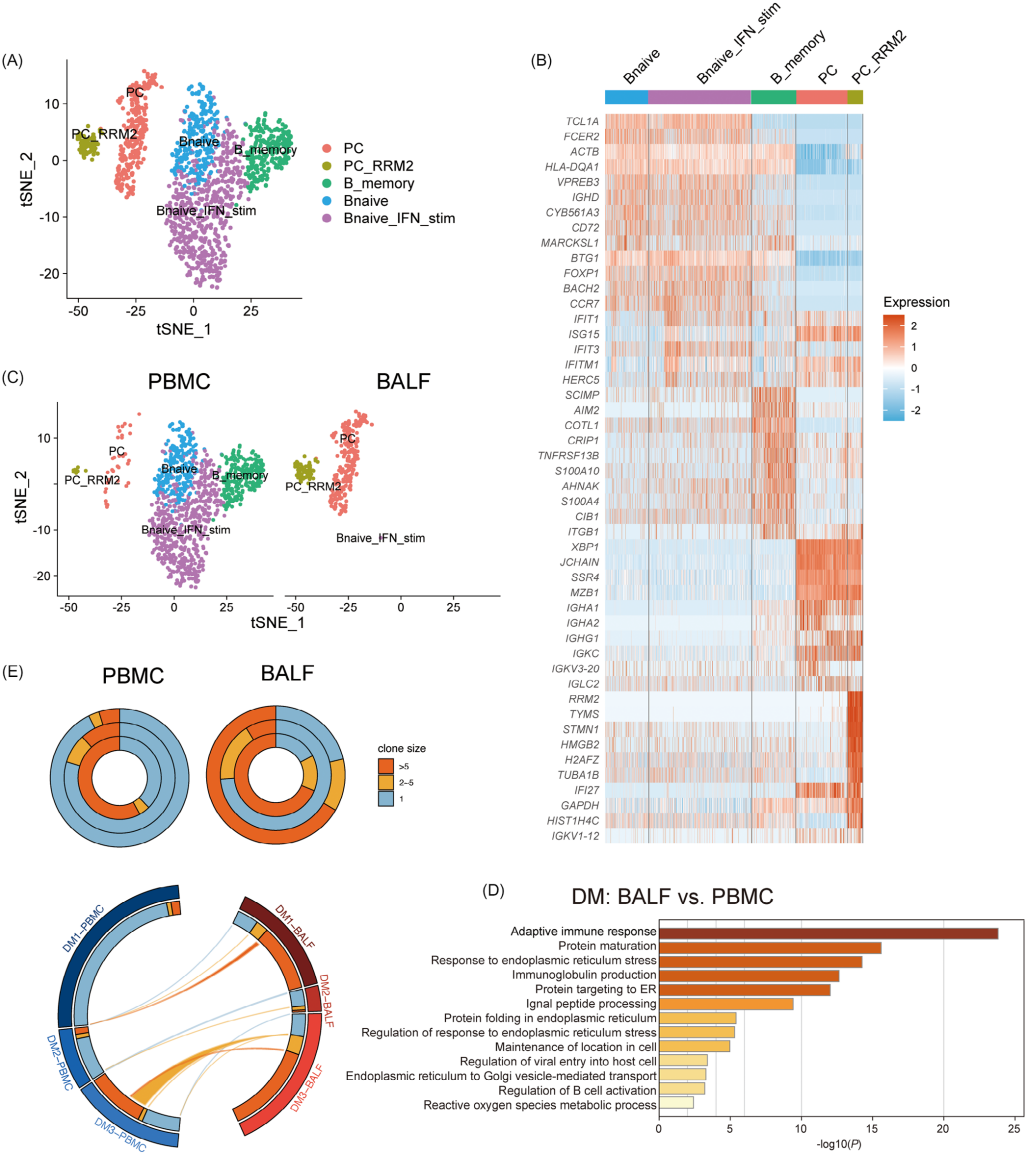
B cell dynamics and abnormal autoantibody responses in BALF of anti‐MDA5+ DM patients. (A) t‐SNE plot illustrating distinct B cell clusters in PBMCs and BALF. (B) Heatmap showing the top 10 marker genes for each B cell cluster. (C) t‐SNE plot comparing B cell clusters between PBMCs and BALF. (D) Bar plot showing enriched GO pathways for upregulated genes in BALF compared with PBMCs. (E) Donut plots (top) illustrating B cell clonal expansion in PBMCs and BALF, with rings representing individual patients. The circus plot (bottom) depicts BCR clonotype sharing between PBMCs and BALF.

Anti‐MDA5+ DM is characterised by the dysregulated autoantibody response, with anti‐MDA5 antibody titres linked to poor prognosis.^32^ To better understand the role of abnormal B cell response in this process, we subclustered them into five subsets according to canonical B cell markers: B naïve cells, B naïve_IFN_stimulated (B naïve_IFN_stim) cells, B_memory cells, PCs and PC_RRM2 (Figures 6A,B and S6A). In BALF samples of DM4 and DM5, we could hardly capture any B cells, so we only present the results for DM1‐3. Notably, plasma B cells were found to be more abundant in BALF than in PBMCs (Figures 6C and S6B), suggesting their potential role in autoantibody production. The functional analysis of upregulated genes in BALF B cells, as opposed to those in PBMCs, showed a particular enrichment in protein complex assembly and protein‐transport‐related pathways, likely reflecting the heightened synthesis of immunoglobulins in these cells (Figure 6D).

Furthermore, we assessed the B cell response by analysing BCR repertoires (Figure 6E). This analysis revealed a heterogeneous distribution of BCR clonal sizes among patients. For instance, one patient showed no clonal expansion in either peripheral or BALF B cells, while another exhibited significant clonal expansion in both compartments. Additionally, a third patient demonstrated increased clonal expansions in BALF B cells. This variability indicates that the humoral immune response of B cells is not spatially confined and can occur both in the lung and periphery. However, these findings, derived from a limited number of patients, necessitate further investigation to fully elucidate the dynamics of BCR repertoires in anti‐MDA5+ DM.

## 3 DISCUSSION

Our study reveals critical insights into the pathogenesis of severe anti‐MDA5+ DM‐ILD through a comprehensive analysis of peripheral and pulmonary immune landscapes using single‐cell RNA sequencing. We demonstrated a stark contrast between peripheral immune suppression and pulmonary immune hyperactivation, driven by monocytes transitioning from an immunosuppressive state in blood to a proinflammatory role in the lung. This highlights the dynamic interplay between peripheral and pulmonary immunity and the pivotal role of the monocyte–macrophage lineage, offering new perspectives for therapeutic targeting.

Our findings corroborate previous reports of elevated cytokines and chemokines, such as IL‐6, as indicators of disease severity in anti‐MDA5+ DM‐ILD.^33^ Patients with RP‐ILD show elevated levels of IL‐15, IL‐1RA, CXCL10, VCAM‐1 and ferritin, further supporting the role of systemic inflammatory markers in reflecting disease progression and outcomes.^34^ Our study adds new insights by emphasising the distinct localised hyperinflammatory response in the lungs. Immune cells in BALF, particularly macrophages, demonstrated robust cytokine and chemokine production, whereas, PBMCs showed no significant upregulation of inflammatory genes. This underscores the lungs as the central site of inflammation in anti‐MDA5+ DM‐ILD, with systemic markers in serum reflecting spillover effects rather than the primary drivers of disease. Among these markers, CCL2 emerged as a key player, and its serum levels strongly correlate with disease progression. This aligns with its well‐known role in recruiting monocytes and amplifying inflammatory cascades, emphasising its potential as both a biomarker and a therapeutic target. Additionally, prior reports have identified high CX3CL1 expression in lung vascular endothelium and its potential role in attracting CX3CR1+ cells to the lungs.^35^, ^36^ In line with this, we observed a rise in *CX3CR1* expression in NK cells and increased levels of *CCR7* in T cells, supporting the hypothesis that immune cell migration to the pulmonary microenvironment contributes to localised inflammation.

A deeper analysis of inflammatory signals highlighted the pivotal role of the monocyte–macrophage system in this context (Figure 3). Specifically, monocyte–macrophages in the BALF of patients, predominantly composed of recruited monocytes and Mo‐AMs, are major contributors to cytokine and chemokine production. As monocytes evolve into Mo‐AMs, there is an observed upregulation of profibrotic genes, implicating their potential role in lung fibrosis development. However, establishing a definitive link between Mo‐AM populations and fibrotic responses requires further in‐depth investigation. Ideally, such research would use lung tissue samples to investigate mesenchymal cell expansion and activation, including myofibroblasts and fibroblasts, and their interactions with Mo‐AMs.

Prior studies have linked reduced peripheral monocyte counts to disease severity^30^ and described a monocyte phenotype characterised by antiviral activity and an overactive IFN response,^17^, ^37^ which we also observed in our findings (Figure 4A). Contrastingly, circulating monocytes in our study were featured with a suppressive phenotype resembling monocytic MDSCs, which are known to expand in various inflammatory conditions.^31^ The expansion, potentially induced by factors such as elevated G‐CSF, GM‐CSF, VEGF and IL‐6,^38^ may reflect immune hyperactivation and excessive inflammatory mediators in anti‐MDA5+ DM‐ILD. To rule out the influence of immunosuppressive therapy, we integrated external data from five treatment‐naïve patients, further confirming that the expansion of MDSC‐like monocytes is intrinsic to anti‐MDA5+ DM‐ILD. MDSCs suppress T cells by producing arginase 1, which degrades l‐arginine and impairs T cell proliferation.^39^, ^40^ They can also induce T cell apoptosis through PD1–PDL1 interactions,^41^ a pathway relevant in anti‐MDA5+ DM where PD1^high^ follicular T helper cells are increased.^42^ Additionally, MDSCs inhibit NK cell activity by producing adenosine.^43^ It has been reported that NK cells significantly decreased and showed inhibitory phenotype in anti‐MDA5+ DM patients.^19^ Our data indicate that peripheral T and NK cells not only decrease in number but also exhibit enrichment in programmed cell death pathways, raising the question of whether MDSC‐like monocytes contribute to lymphopenia and immune cell death in this condition. Future studies could explore whether CD14+ HLA^low^ monocytes in anti‐MDA5+ DM can suppress other immune cells and identify the key pathways involved. Excessive cell death may release intracellular components, such as the MDA5 protein, which may serve as autoantigens and lead to the generation of autoantibodies. Taken together, the decrease of T and NK cells and the increase of MDSC‐like monocytes suggest a state of peripheral immune suppression.

Building on the findings from peripheral monocytes and pulmonary macrophages, an important question arises: what connections link these two cell populations? We propose that CD14+_HLA^low^ monocytes are recruited to the lung via chemokine‐mediated pathways and subsequently differentiate into proinflammatory Mo‐AMs within the pulmonary microenvironment. The CD14+_HLA^low^ monocyte cluster, which is nearly absent in HCs but constitutes over 75% of peripheral monocytes in anti‐MDA5+ DM patients, suggests a significant role in disease progression. Elevated expression of *CCR1* and *CCR2* further supports the hypothesis that these monocytes are preferentially recruited to the lung. Within the pulmonary microenvironment, these cells undergo further activation, acquiring a highly proinflammatory phenotype characterised by calprotectin expression (*S100A8, S100A9, S100A12*). This observation aligns with previous reports that MDSCs exhibit proinflammatory functions in autoimmune diseases. In SLE, MDSCs promote Th17 differentiation and exacerbate disease severity,^44^, ^45^ while in RA, they produce proinflammatory cytokines (e.g., TNF‐α, IL‐1β) and induce Th17 differentiation.^46^, ^47^, ^48^ Furthermore, MDSCs exhibit notable plasticity, with their differentiation and function varying depending on organ‐specific microenvironments.^49^ For instance, in liver cancer, MDSCs predominantly exert immunosuppressive activity to promote tumour immune evasion, whereas in lung cancer, they differentiate into M1‐like proinflammatory macrophages and contribute to inflammation. Similarly, in anti‐MDA5+ DM, CD14+_HLA^low^ monocytes likely undergo a proinflammatory transformation, amplifying local immune responses in the lungs. However, the functional and migratory relationships between peripheral CD14+_HLA^low^ monocytes and Mo‐AMs require further investigation. Future studies using lineage‐tracing animal models and integrative multi‐omics approaches are essential to clarify their roles in disease pathogenesis. Despite the absence of mechanistic investigations in our study, the presented data underscore the significant role of recruited monocytes and Mo‐AMs in the pathogenesis of anti‐MDA5+ ILD. Consequently, targeting monocyte recruitment and macrophage activation presents a promising therapeutic strategy. Specifically, CCR2 antagonist or CCL2‐neutralising antibodies could be employed to block monocyte recruitment. CCR2 inhibitors (e.g., PF‐04136309^50^ and CCX872^51^), have demonstrated efficacy in reducing tumour‐associated macrophage infiltration in cancer models and may offer similar benefits in limiting lung inflammation in this context. Furthermore, strategies to modulate macrophage phenotype should focus on reducing both proinflammatory (M1) and profibrotic (M2) activities. The M1 phenotype is primarily driven by the signal transducer and activator of transcription 1 (STAT1), whereas, STAT6 regulates M2 polarisation.^52^ Tofacitinib, a JAK inhibitor targeting JAK1 and JAK3, modulates downstream STAT pathways, thereby suppressing the activities of both M1 and M2 macrophages.^53^ Notably, prior studies have reported the superior efficacy of combination therapy with calcineurin inhibitors (CNIs) and tofacitinib than CNIs alone,^18^ highlighting the potential of targeting macrophage function alongside broader immune pathways for improved disease control.

Epidemiological studies have suggested that viral infections might trigger anti‐MDA5+ DM, evidenced by its seasonal and geographical patterns.^54^, ^55^ Emerging links between anti‐MDA5+ DM and SARS‐CoV‐2 further support this hypothesis. Reports of anti‐MDA5 antibodies and associated symptoms following COVID‐19 infection or vaccination,^56^, ^57^ point to a potential immunological response that mimics or induces anti‐MDA5+ DM pathology. Notably, shared features are observed between anti‐MDA5+ RP‐ILD and COVID‐19, such as proinflammatory Mo‐AM infiltration,^58^ decreased HLA‐DR expression on CD14+ monocytes,^59^, ^60^ and distinct peripheral‐lung immune responses.^61^ These similarities could be key in unravelling the pathogenesis of ILD, whether it emerges in the context of anti‐MDA5+ DM or as a consequence of viral infections like COVID‐19.

In a previous single‐cell resolution study of adaptive immune cells in anti‐MDA5+ DM, Ye et al.^18^ found enhanced peripheral antibody‐secreting cell and CD8 T cell response. Another recent study performing scRNA‐seq analysis on PBMCs of anti‐MDA5+ DM reported the antiviral response and activation of IFN signalling in both innate and adaptive immune cells.^37^ Although these two studies have uncovered several aspects of the anti‐MDA5+ DM pathogenesis, a panoramic view involving the pulmonary immune landscape remains lacking. By integrating scRNA‐seq analysis of paired BALF and PBMCs, our study bridges this gap, highlighting the intricate immune crosstalk between peripheral and pulmonary compartments and advancing the systemic understanding of anti‐MDA5+ associated ILD immunopathogenesis.

Our study faced several limitations. First, the significant heterogeneity within anti‐MDA5+ DM, and the existing subtyping proposed by other research,^62^, ^63^ was not mirrored in our approach as we did not differentiate between severe and non‐severe cases, nor did we conduct longitudinal follow‐up studies to observe treatment responses over time. Second, although our findings highlight the critical role of the monocyte–macrophage system in inflammation, the precise origin of this inflammatory response remains to be clarified, necessitating more detailed sequential research. Third, our use of HC data from public databases, despite matching for ethnicity, age and gender, may have introduced biases.

In summary, our scRNA‐seq dataset has uncovered various previously underappreciated immune characteristics of anti‐MDA5+ DM, highlighting the contrasting immune landscapes of peripheral immune suppression alongside pulmonary immune hyperactivation. Central to this dysregulation is the monocyte–macrophage lineage, which emerges as a key driver of disease pathology. This enhanced understanding of the disease's pathogenesis opens new avenues for the identification of novel therapeutic targets and refinement of clinical assessment strategies in anti‐MDA5+ DM.

## 4 METHODS

### 4.1 Study design

This study aimed to gain insights into the disrupted immune landscape spanning both the peripheral and pulmonary domains in individuals afflicted with severe anti‐MDA5+ DM and identify potential therapeutic targets in anti‐MDA5+ DM. We employed single‐cell RNA sequencing to examine cellular constituents within patients’ BALF and paired PBMCs. Luminex assay and flow cytometry were used to further validate the findings. Peripheral blood and BALF samples were collected from patients meeting the 2017 EULAR/ACR Classification Criteria for Idiopathic Inflammatory Myopathies.^64^ All subjects provided written informed consent.

### 4.2 Sample preparation

PBMCs were isolated from blood using density gradient centrifugation and subsequently resuspended in phosphate‐buffered saline (PBS).

The collection of BALF is performed by a qualified and experienced physician with specialised training in an intensive care unit (ICU). The operation follows the clinical practice guidelines of the American Thoracic Society from 2012.^65^ The most affected lung segment, as identified on HRCT, is selected for the lavage, which is performed under local anaesthesia. Normal saline at room temperature (100–300 mL, divided into 3–5 aliquots) was instilled through the bronchoscope. A minimum of 5% of the instilled volume was retrieved. Fresh BALF was filtered through a 100‐µm nylon cell strainer to remove aggregates and debris, then centrifuged. The resulting cell pellets were resuspended in chilled RPMI 1640 complete medium. The supernatant was collected for cytokine detection. Both BALF and PBMC samples were analysed fresh.

### 4.3 MDSC and ncMono Flow cytometry

PBMC samples for flow cytometry were collected during routine follow‐up visits (every 3–6 months), with most patients in remission and a small subset experiencing relapse. For cell‐surface labelling, 1 × 10^6^ cells were blocked with Fc‐block reagent (BD Biosciences) and True‐Stain Monocyte Blocker (BioLegend). The following antibodies were then added and incubated for 30 min: CD14‐PE (BioLegend; 63D3), HLA‐DR‐APC‐Cy7 (BioLegend; L243), CD3‐APC (BioLegend; HIT3a), CD8‐APC (BioLegend; RPA‐T8), CD19‐APC (BioLegend; HIB19), CD56‐APC (BioLegend; HCD56) and CD16‐FITC (BioLegend; 3G8). To distinguish live from dead cells, the cells were incubated with 7‐AAD (Biogems; 61410‐00). After incubation, the samples were washed and resuspended in PBS for flow cytometric analysis using a BD FACSAria II. CD14 and HLA‐DR were used to gate MDSCs, while CD3, CD8, CD19, CD56, CD14 and CD16 were used to gate ncMonos.^66^

### 4.4 Cell death detection by flow cytometry

Cell death was assessed using the Annexin V APC Apoptosis Detection Kit (Biogems; 62700) according to the manufacturer's instructions. Briefly, cells were incubated with Annexin V‐APC and 7‐AAD in 1× Annexin V binding buffer for 15 min at room temperature. After staining, 400 µL of 1× binding buffer was added, and the samples were analysed on a BD FACSAria II.

### 4.5 Cytokine detection

Cytokines in the serum or BALF supernatant were detected using the multiplexed Luminex xMAP assay (eBioscience) following the manufacturer's instruction. To study inflammation in BALF and serum, we selected cytokines based on their established roles as key drivers of inflammation, ensuring a focused analysis of immune and inflammatory dynamics.

### 4.6 Single‐cell RNA sequencing

Single‐cell RNA‐seq libraries were prepared using the Chromium Single Cell 3ʹ Reagent Kits v3 (10× Genomics), following the manufacturer's protocol. Gene expression libraries were sequenced on a NovaSeq platform (Illumina) to generate 150 bp paired‐end reads.

### 4.7 Processing of scRNA‐seq data

The datasets of HCs are from GSE158055 (HC003, HC005, HC008 and HC010 were chosen as controls for PBMCs) and GSE14592 (HC1, HC2 and HC3, were chosen as controls for BALF). All single‐cell transcriptome sequencing data were aligned and quantified using Cell Ranger (v6.0.2) with the GRCh38 human reference genome obtained from the 10× Genomics official website. The filtered feature matrixes were then imported into Seurat (V 4.1.2) for quality control. All functions were executed with default parameters unless specified otherwise. We retained cells with between 300 and 5000 expressed genes and a mitochondrial percentage of less than 10%. Principal component analysis (PCA) was performed using the 2000 most variable features identified by the *FindVariableFeatures*. The PCA matrix was then imported into Harmony (V0.1.1, R) to integrate the single‐cell gene expression data and correct for batch effects.

### 4.8 Cell clustering and annotation

UMAP was performed on the top 20 principal components to visualise the cells. The first round of unsupervised clustering (resolution = 0.5) was used to identify the main cell types, including the NK cells, CD4 T cells, CD8 T cells, B cells, PCs, monocytes, DCs, platelets, macrophages and epithelial cells. To further define subclusters within each major cell type, a second round of clustering was performed separately on T cells, NK cells, B cells, monocytes and macrophages (resolution = 1).

### 4.9 Differential gene expression and pathways enrichment analyses

FindMarkers function (Seurat) was used to identify DEGs (|Log2FoldChange | > 1, adjusted *p *< .05). To investigate the functions of these DEGs, we performed GO pathway enrichment analysis using ClusterProfiler (v4.6.0) in R. Enrichment analysis of the B cell part was conducted using the Metascape web tool (www.metascape.org).

### 4.10 Inflammatory score, cytokine score, MHC class II score, calprotectin protein score, MDSC‐like score, M1 score and M2 score

Scores were calculated using the AddModuleScore function in the Seurat package. Inflammatory score, cytokine score,^21^ MHC class II score, calprotectin protein score and MDSC‐like score^61^ were calculated according to published literature. The reference genes used for calculating these scores are provided in Table S4.

### 4.11 Pseudo‐time trajectory analysis

Trajectory analysis for monocyte–macrophages was performed with slingshot.^67^ To integrate monocyte–macrophage data from PBMCs and BALF, cell trajectories were inferred with peripheral monocytes as the starting point.

### 4.12 Calculation of gene expression program scores

To assess the transcriptional similarity between macrophages in our dataset and those from previously published pulmonary fibrosis studies, we used gene set module scoring. Specifically, gene expression program scores were calculated using the AddModuleScore function in Seurat (seed = 1993). Reference gene sets were derived from three published pulmonary fibrosis datasets.^23^, ^25^, ^68^ For each reference dataset, the top 50 marker genes were used to define the module. In cases where fewer than 50 genes were available, all genes from the respective reference were included. This approach allowed for the quantification of transcriptional profiles indicative of profibrotic macrophage phenotypes, as described in Ref. ^69^.

### 4.13 BCR and TCR repertoire analysis

On the basis of TCR and BCR CDR3 amino acid sequences, we compared the clone size distribution and clonotype in the periphery and BALF of the same patient.

### 4.14 Histological analysis

The samples of lung tissue were fixed in 4% formaldehyde and embedded in paraffin (FFPE). FFPE blocks were sectioned into 4 µm slices, deparaffinised, rehydrated and stained with haematoxylin–eosin and Masson's trichrome following standard protocols.

### 4.15 Statistics

Quantitative data were expressed as the mean ± standard error of the mean. Statistical analysis was performed by Prism 9.0 (GraphPad). For comparisons between two groups, independent‐sample *t*‐tests or paired *t*‐tests were used for normally distributed variables and Mann–Whitney tests or Wilcoxon rank‐sum tests were applied for non‐normally distributed variables. For comparisons of more than two groups, the Kruskal–Wallis test was used for data not following a normal distribution. Spearman's correlation was applied for correlation analyses. All statistical tests were two‐tailed, with a *p* value < .05 considered significant.

## AUTHOR CONTRIBUTIONS

*Conceptualisation*: Jun Liu, Houzao Chen and Qian Wang *Patient information acquisition*: Chanyuan Wu, Jinmin Peng, Shuang Zhou, Dong Xu, Jiuliang Zhao, Bin Du and Chen Yao *Data analysis and interpretation*: Jia Shi, Xiaoya Pei, Yangzhong Zhou, Yulin Lv and Xiaoman Wang *Validation*: Jia Shi, Xueting Yuan and Xingbei Dong *Manuscript writing, review, and editing*: Jia Shi, Xiaoya Pei, Yangzhong Zhou, Qian Wang and Jinmin Peng *Supervision*: Yulin Lv, Jinmin Peng and Xiaofeng Zeng *Funding acquisition*: Mengtao Li, Qian Wang and Chanyuan Wu. All authors agreed to submit the manuscript, have read and approved the final draft, and take full responsibility for its content, including the accuracy of the data and their statistical analysis.

## CONFLICT OF INTEREST STATEMENT

The authors declare no conflicts of interest.

## ETHICS STATEMENT

Ethical approval was obtained from the Research Ethics Committee of Peking Union Medical College Hospital (JS‐2038) and Chinese PLA (People's Liberation Army) General Hospital (2022052701006).

## Supporting information



Supporting Information

Supporting Information

Supporting Information

Supporting Information

Supporting Information

Supporting Information

Supporting Information

## Data Availability

The raw sequence data presented in the study are deposited in the Genome Sequence Archive for Human, accession number HRA008638 (https://bigd.big.ac.cn/gsa‐human/browse/HRA008638), which are available on reasonable request. The scripts used for all analyses in this study are available on GitHub (https://github.com/peixiaoya/MDA5_project_2023.git).
