# Supplementary figures and images for: Monocyte–macrophage dynamics as key in disparate lung and peripheral immune responses in severe anti‐melanoma differentiation‐associated gene 5‐positive dermatomyositis‐related interstitial lung disease
Source: Clin Transl Med. 2025 Feb 4;15(2):e70226. doi: 10.1002/ctm2.70226 (PMC11791760; doi:10.1002/ctm2.70226)

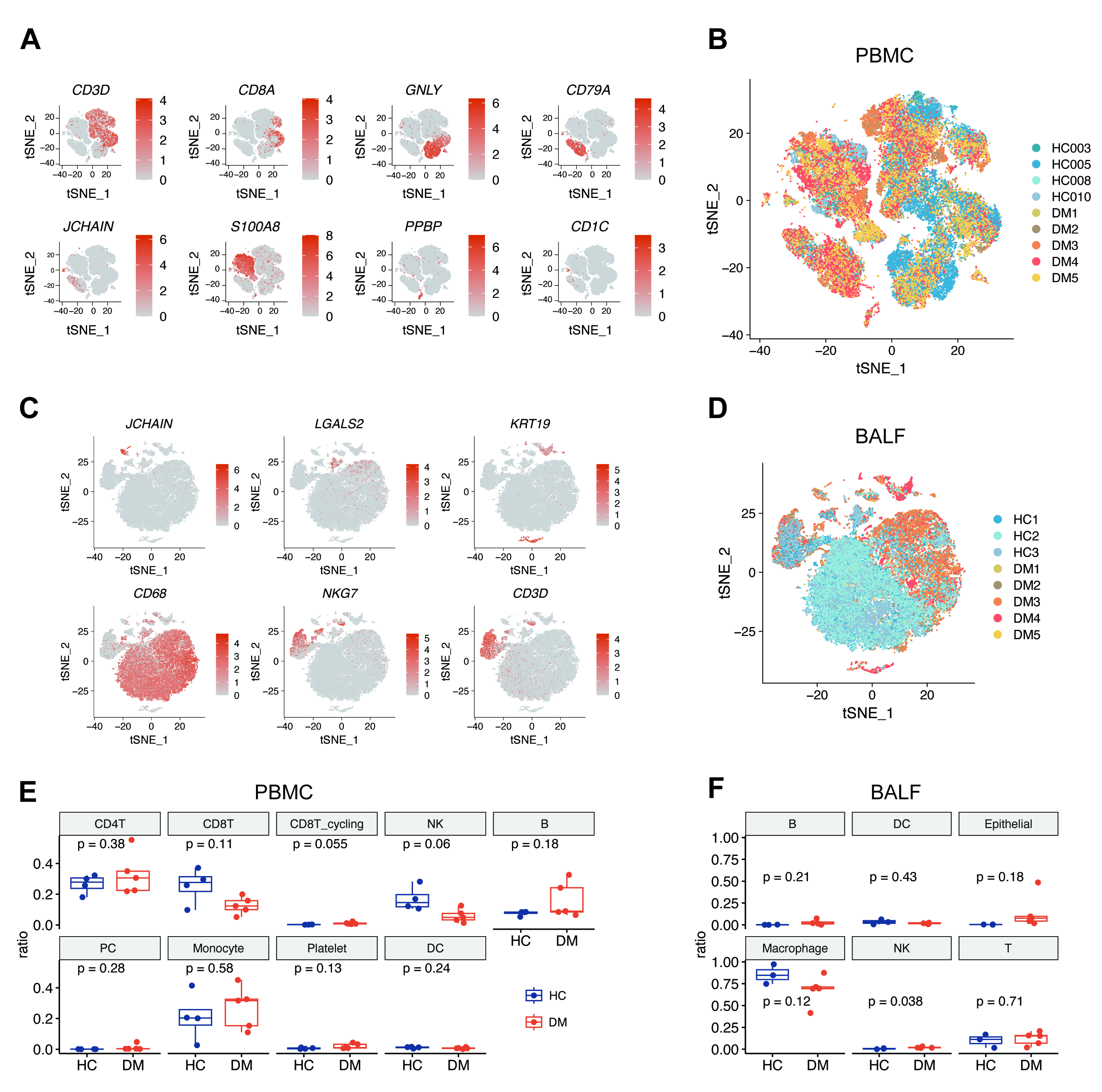

Supplement: Supplementary file 1 — Supporting Information [file CTM2-15-e70226-s007.tif]

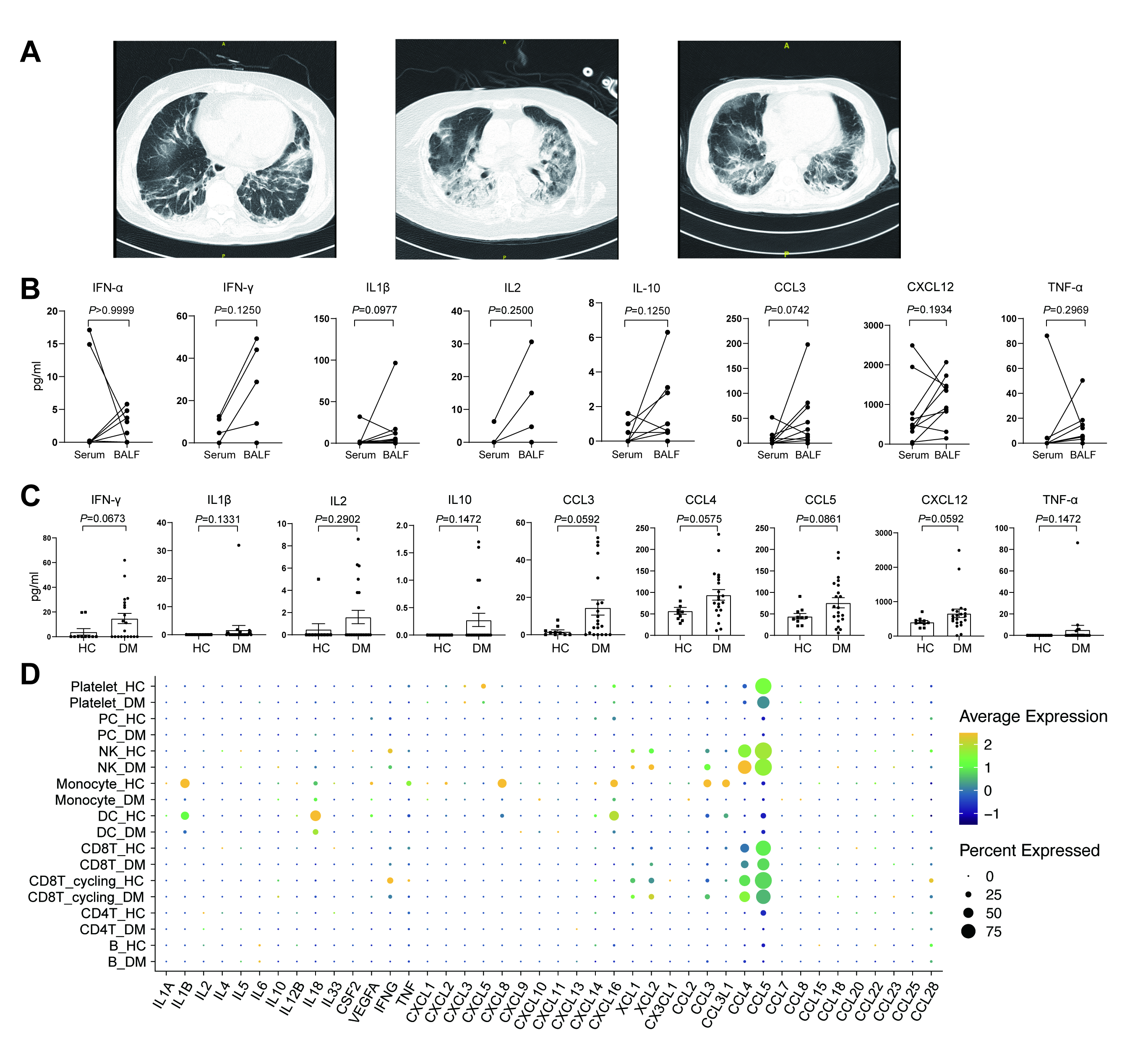

Supplement: Supplementary file 2 — Supporting Information [file CTM2-15-e70226-s001.tif]

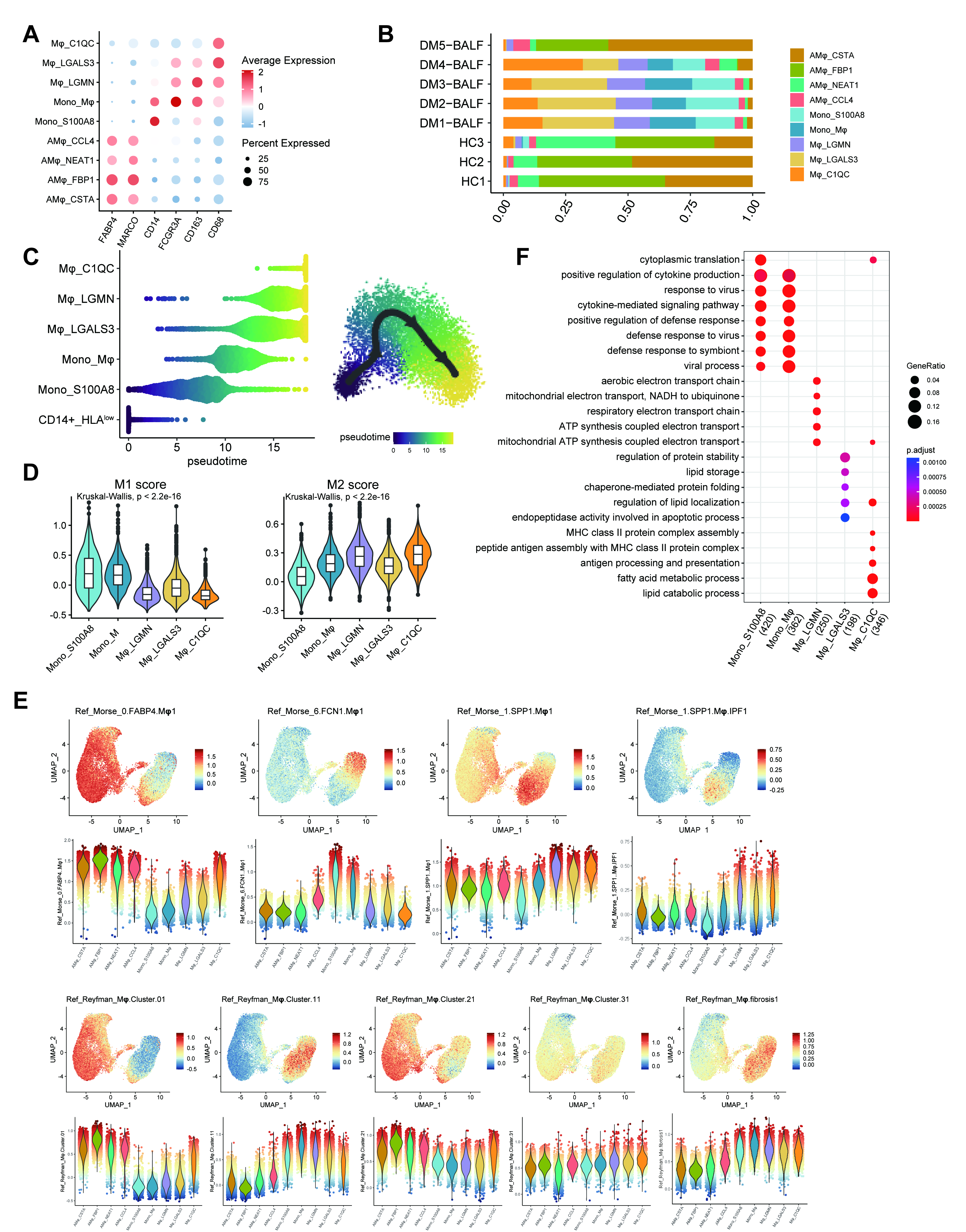

Supplement: Supplementary file 3 — Supporting Information [file CTM2-15-e70226-s005.tif]

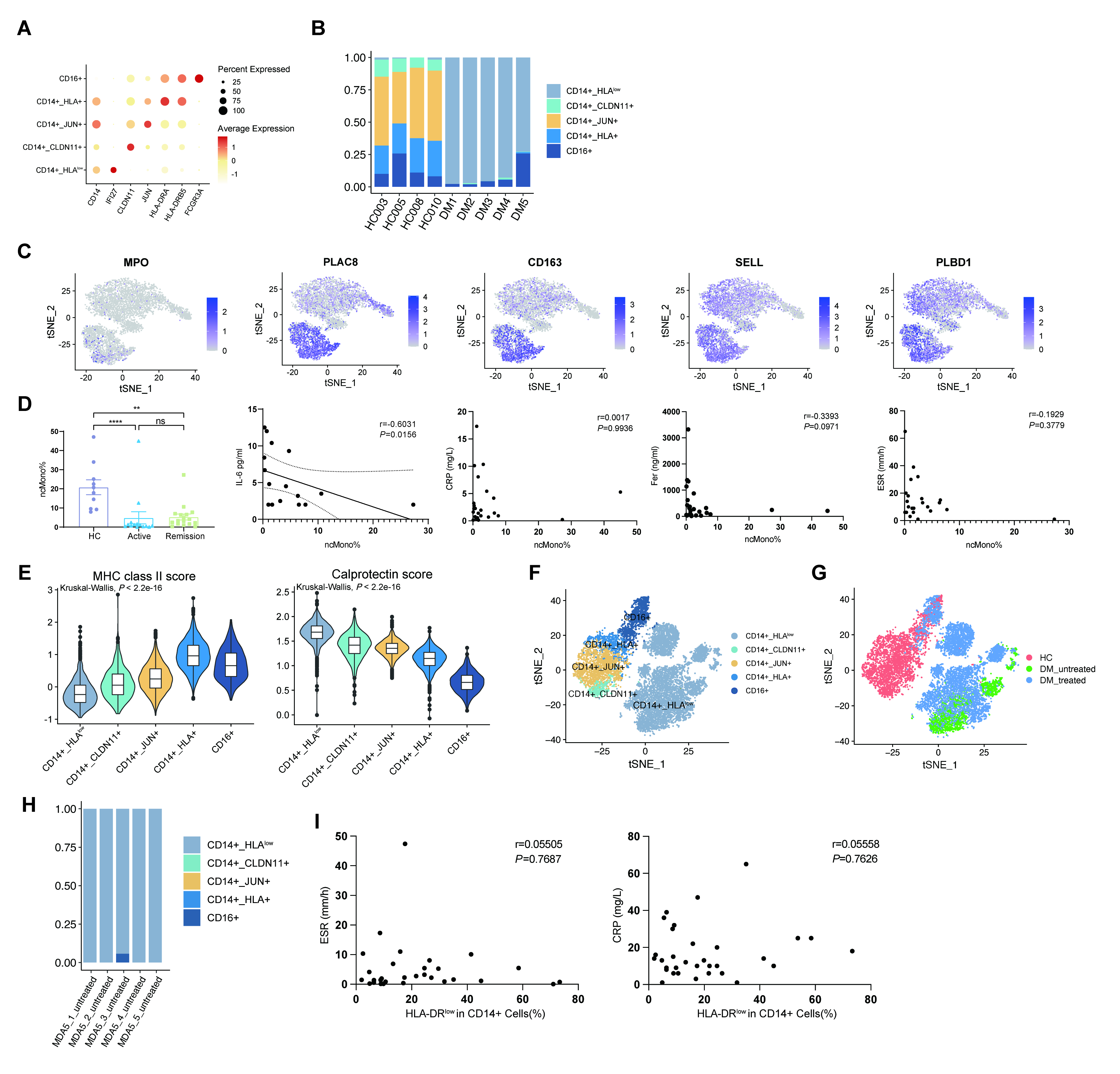

Supplement: Supplementary file 4 — Supporting Information [file CTM2-15-e70226-s006.tif]

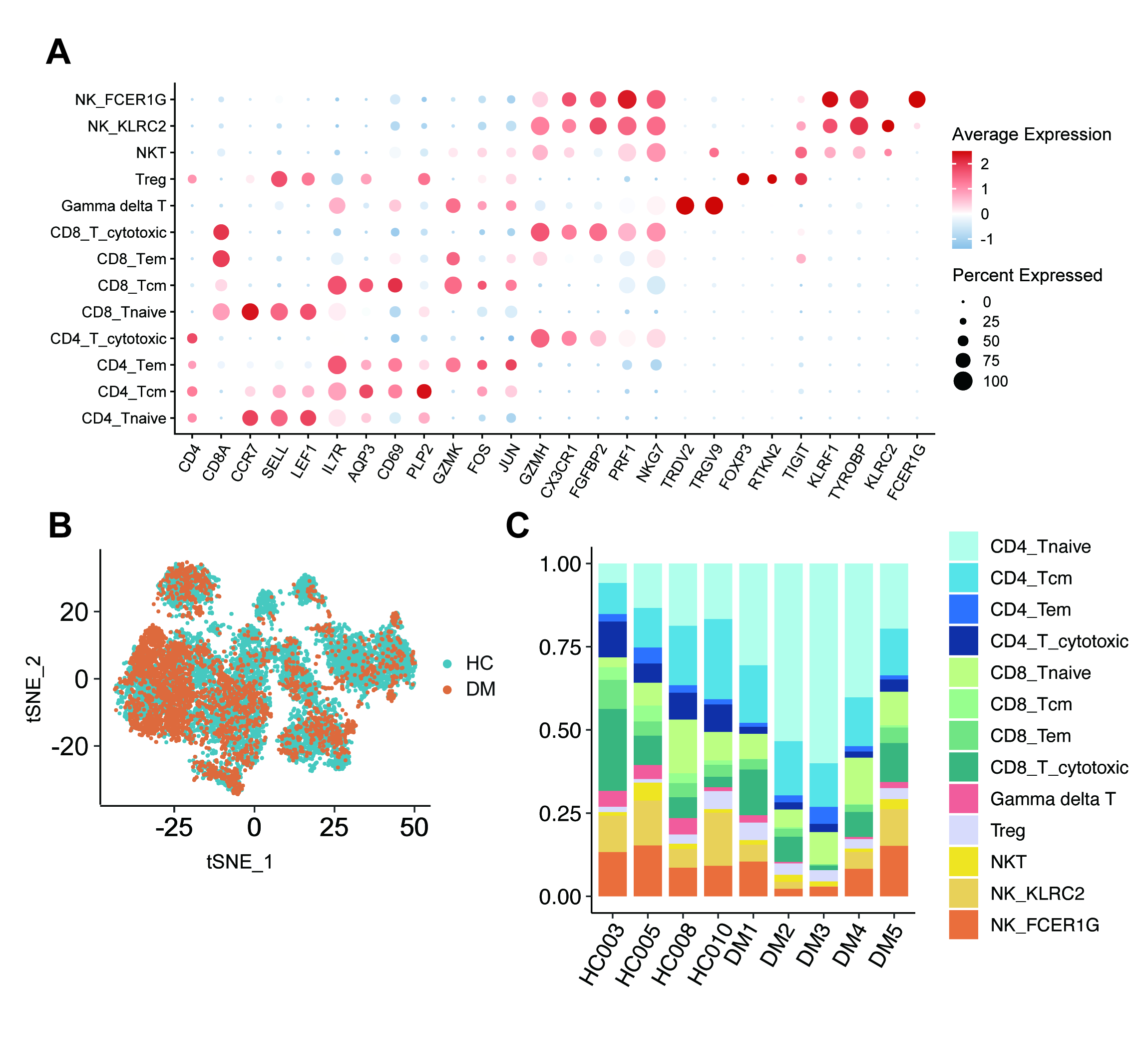

Supplement: Supplementary file 5 — Supporting Information [file CTM2-15-e70226-s003.tif]

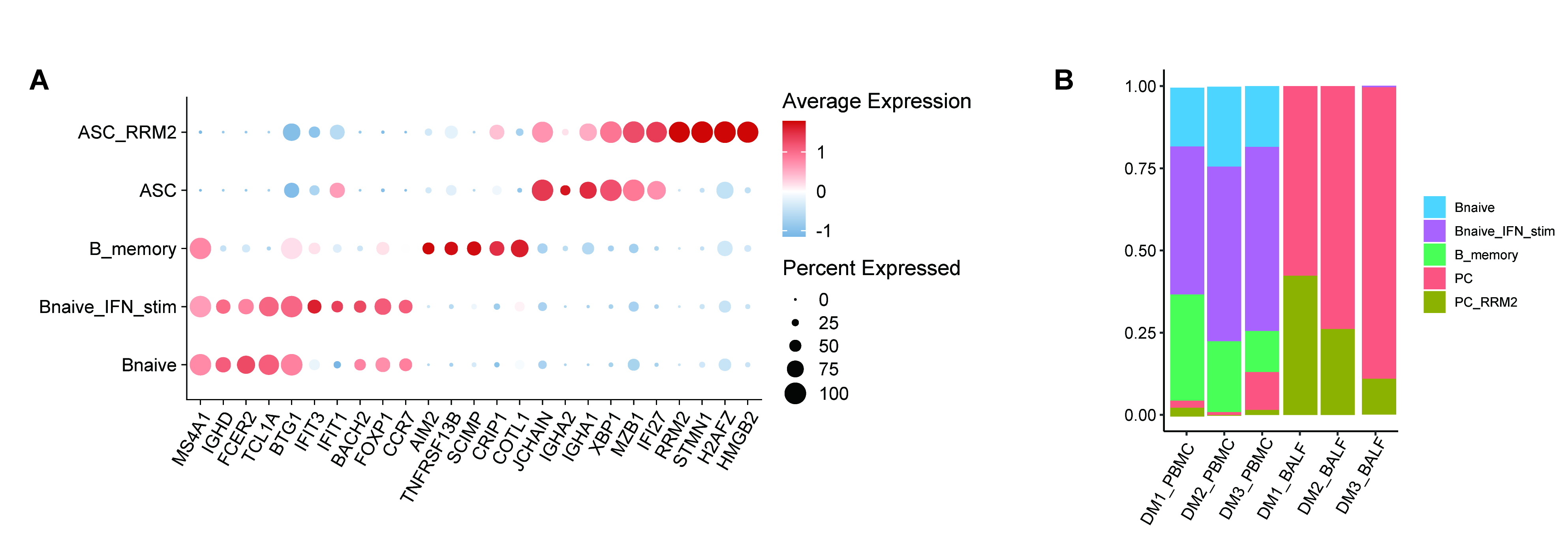

Supplement: Supplementary file 6 — Supporting Information [file CTM2-15-e70226-s002.tif]
