# Supplementary material for: Monocyte–macrophage dynamics as key in disparate lung and peripheral immune responses in severe anti‐melanoma differentiation‐associated gene 5‐positive dermatomyositis‐related interstitial lung disease
Source: Clin Transl Med. 2025 Feb 4;15(2):e70226. doi: 10.1002/ctm2.70226 (PMC11791760; doi:10.1002/ctm2.70226)
Supplement: Supplementary file 7 — Supporting Information [file CTM2-15-e70226-s004.docx]

**Supplemental Materials**

**Supplemental Figures**

**
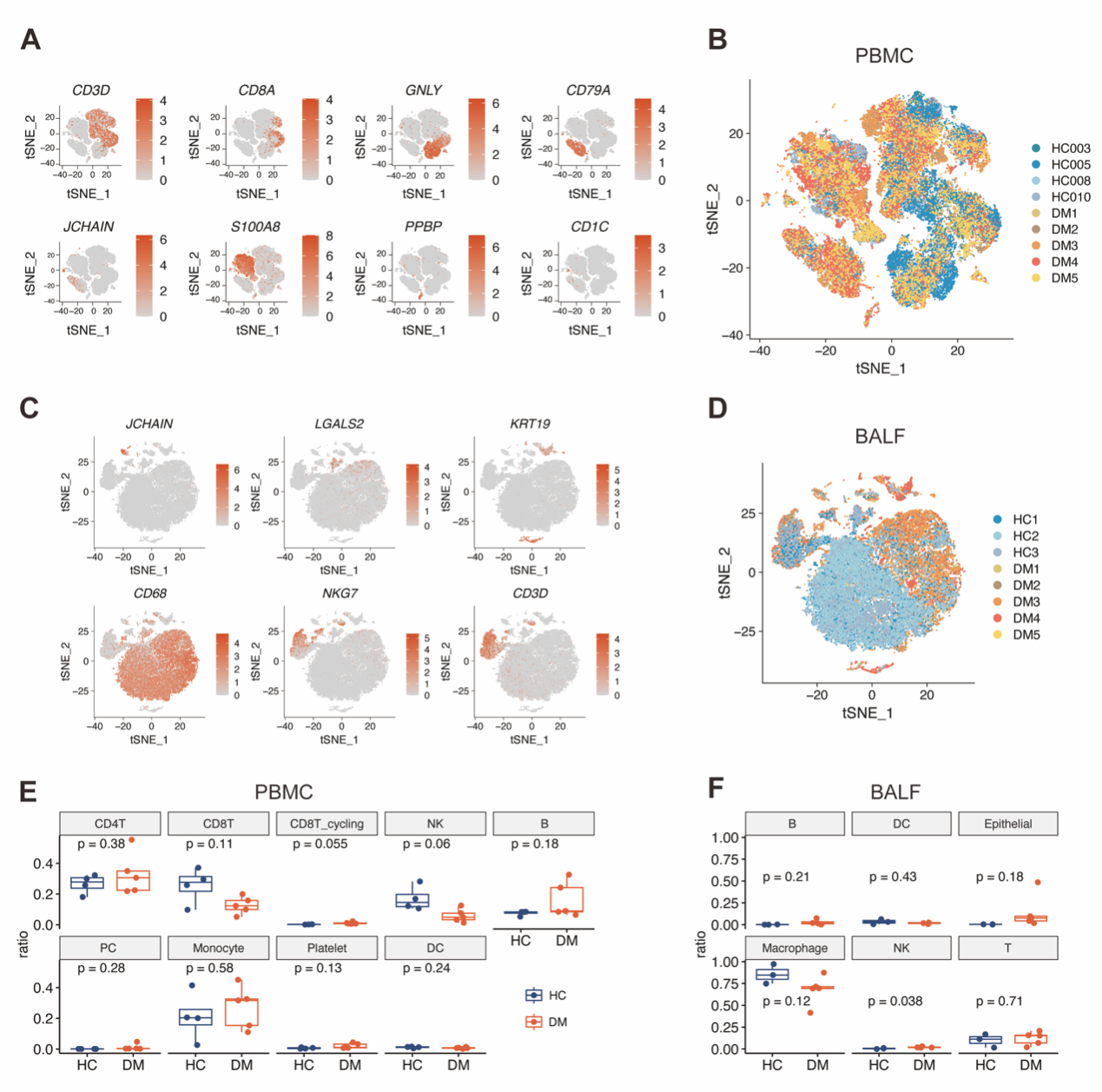
**

**Figure S1 The clustering and annotation data of PBMCs and BALF, related to Figure 1.**

**(A & C)** Expressions of canonical marker genes for major cell types in PBMCs **(A)** and BALF **(C)**, represented on t-SNE plots.

**(B & D)** tSNE plots showing the single-cell transcriptomes of PBMCs **(B)** and BALF **(D)** from different samples. Colors indicate sample groups.

**(E & F)** Boxplots showing the proportions of each cell cluster in PBMC **(E)** and BALF **(F)** for HC and anti-MDA5+ DM groups.


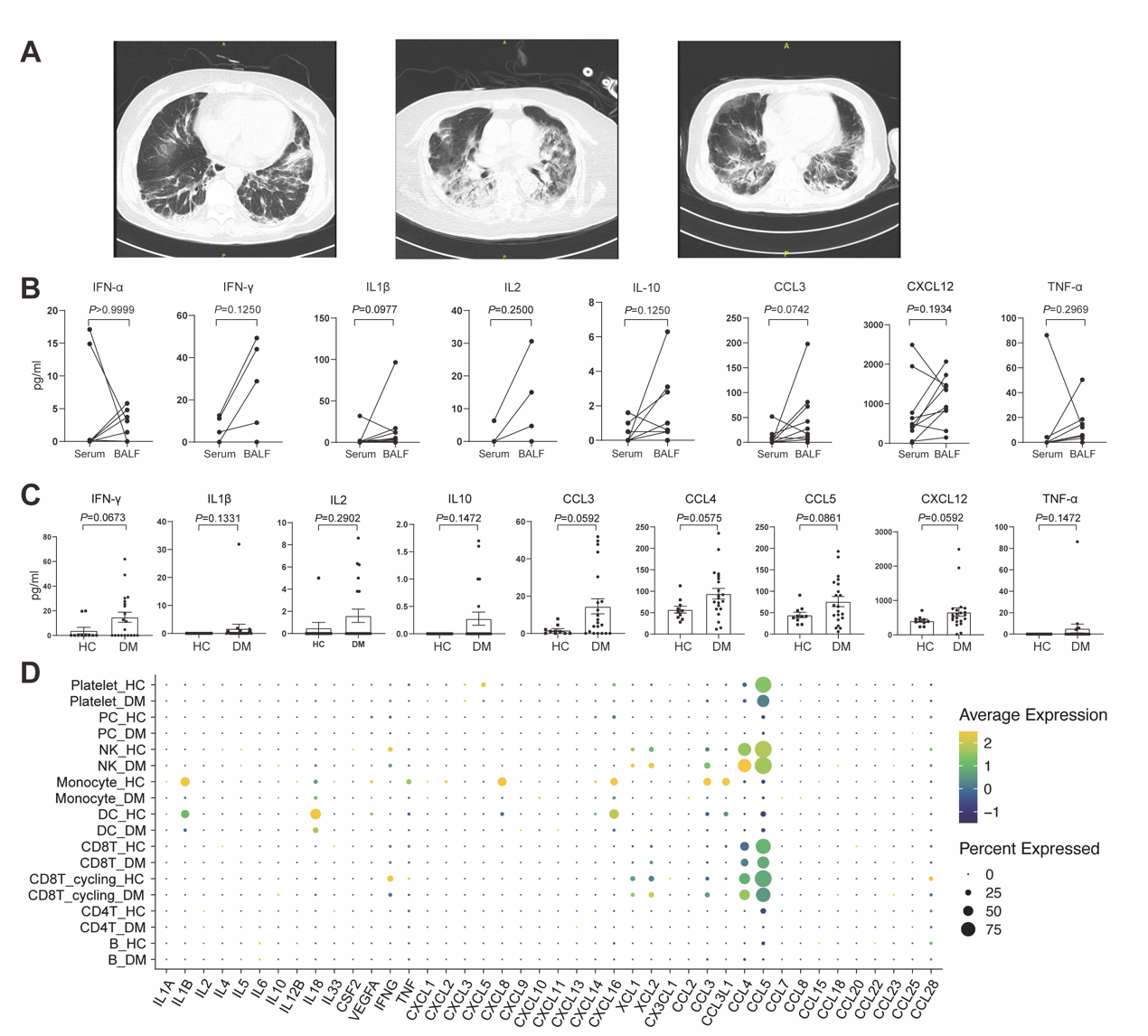


**Figure S2 Cytokine and chemokine levels in circulation and BALF of anti-MDA5+ DM patients, related to Figure 2.**

**(A)** Representative high-resolution computed tomography images from three patients with anti-MDA5+ DM-ILD.

**(B)** Scatter plots showing cytokine and chemokine levels in paired BALF and serum samples of anti-MDA5+ DM patients (n = 10).

**(C)** Scatter plots showing cytokine and chemokine levels in the serum of anti-MDA5+ DM patients (n=20) and HCs (n = 10).

**(D)** Dot plot depicting gene expression levels of cytokines and chemokines in PBMC clusters from anti-MDA5+ DM patients and HCs. Colors represent average expression levels, while dot sizes indicate the percentage of cells expressing the genes within each cluster.


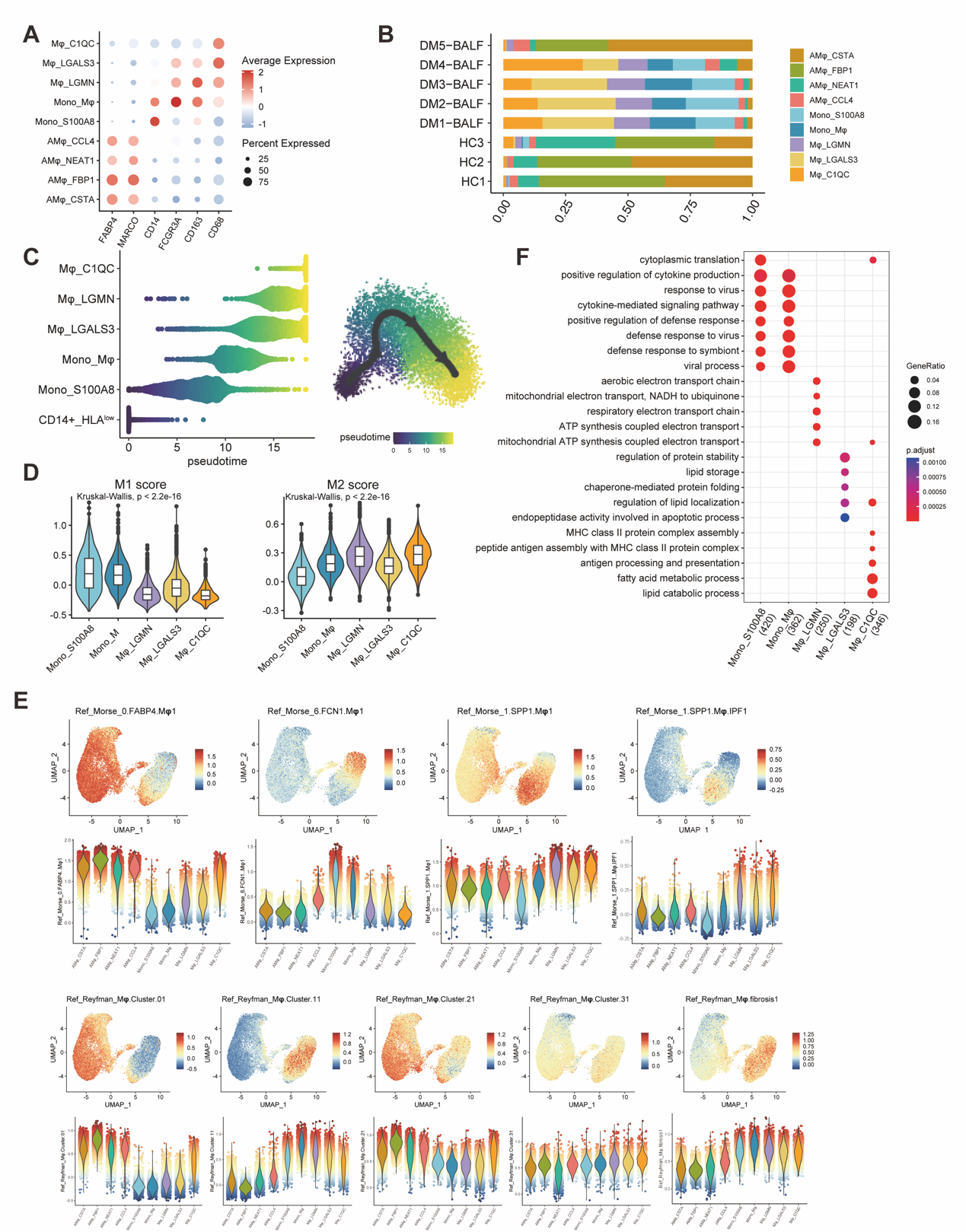


**Figure S3 Monocyte-macrophage lineage analysis in the BALF of anti-MDA5+ DM patients, related to Figure 3.**

1. Dot plot depicting the expression of hallmark genes across distinct monocyte-macrophage clusters in the BALF of anti-MDA5+ DM patients. Dot size represents the percentage of cells expressing the gene, and color indicates the average expression level.
2. Bar plot illustrating the relative proportions of each monocyte-macrophage cluster across individual subjects.

**(C)** Pseudo-time trajectory analysis of monocyte-macrophage lineages in anti-MDA5+ DM patients.

**(D)** Violin plots showing the M1 and M2 polarization scores of each monocyte-macrophage cluster.

**(E)** UMAP visualization (top) and violin plots (bottom) illustrating signature module scores of monocyte-macrophage clusters associated with idiopathic pulmonary fibrosis (IPF) from two publicly available datasets^1,2^. Scores are projected onto BALF monocyte-macrophage clusters, with violin plots color-coded to match cluster identities as shown in **Figure 3A**.

**(F)** Representative Gene Ontology (GO) pathways enriched by the highly expressed genes in monocyte-macrophage subtypes.


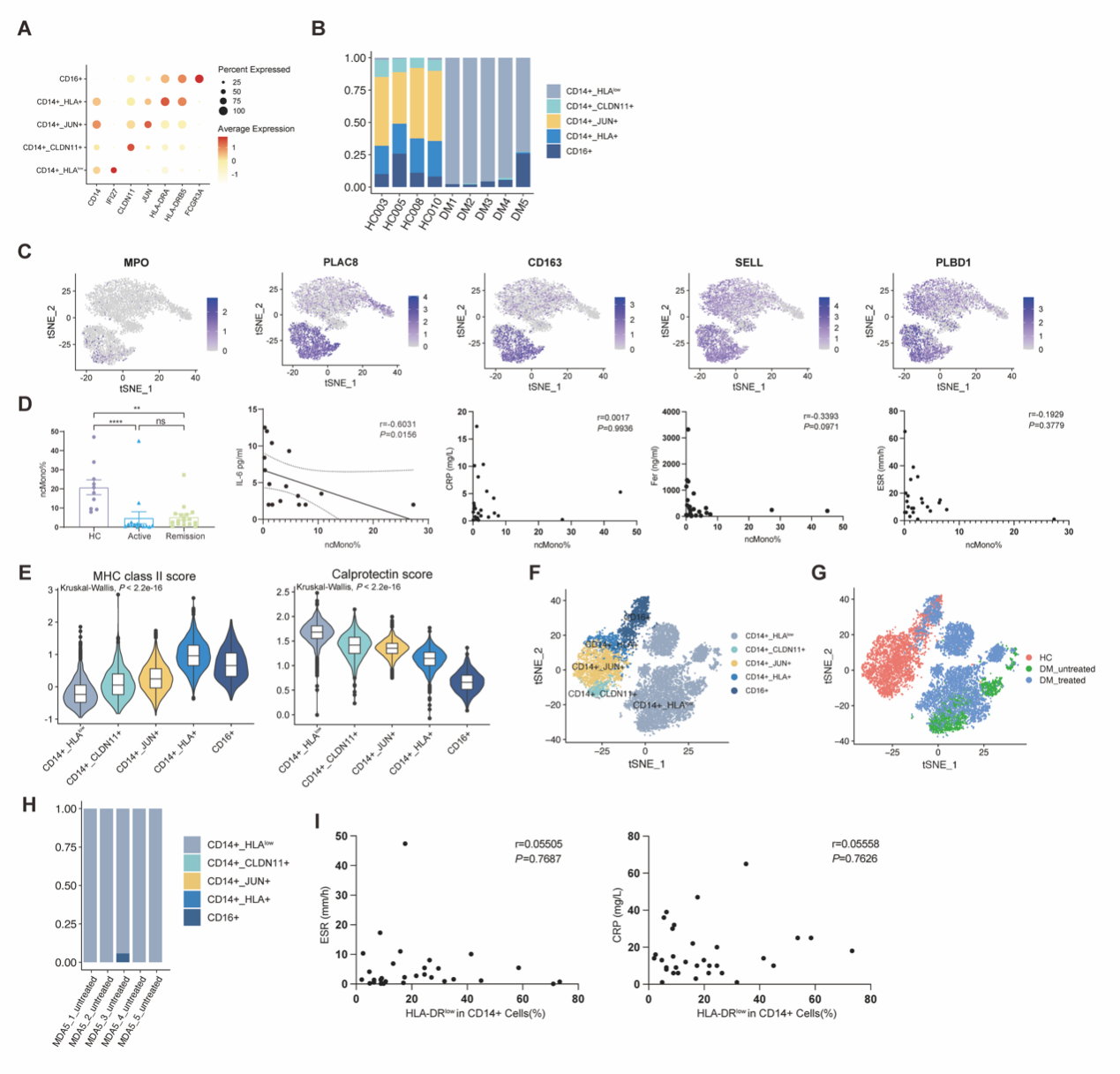


**Figure S4 Analysis of peripheral monocytes in anti-MDA5+ DM, related to Figure 4.**

1. Dot plot showing hallmark gene expression across monocyte clusters in PBMCs.

**(B)** Bar plot depicting the proportions of each monocyte cluster across studied subjects.

**(C)** t-SNE plots showing the expression of selected genes associated with immaturity and anti-inflammatory functions in monocytes.

**(D)** Left: Scatter plot comparing the percentage of non-classical monocytes among CD14+ PBMCs in anti-MDA5+ DM-active (n = 15), anti-MDA5+ DM-remission (n = 18), and HC (n = 10) groups. Right: Spearman correlation analysis of non-classical monocyte percentage with IL-6, CRP, ESR, and ferritin levels.

**(E)** Violin plots showing MHC class II score and calprotectin score for each monocyte cluster.

**(F)** t-SNE plot displaying the five monocyte cell clusters identified in PBMCs after integrating data from five treatment-naïve anti-MDA5+ DM-ILD patients.

**(G)** t-SNE plot displaying the distribution of peripheral monocytes across treated patients, untreated patients, and HCs.

**(H)** Bar plot showing the proportions of monocyte clusters in treatment-naïve patients.

**(I)** Spearman correlation analysis between the percentage of HLA-DR^low^ monocytes and CRP (left), and ESR (right) levels.


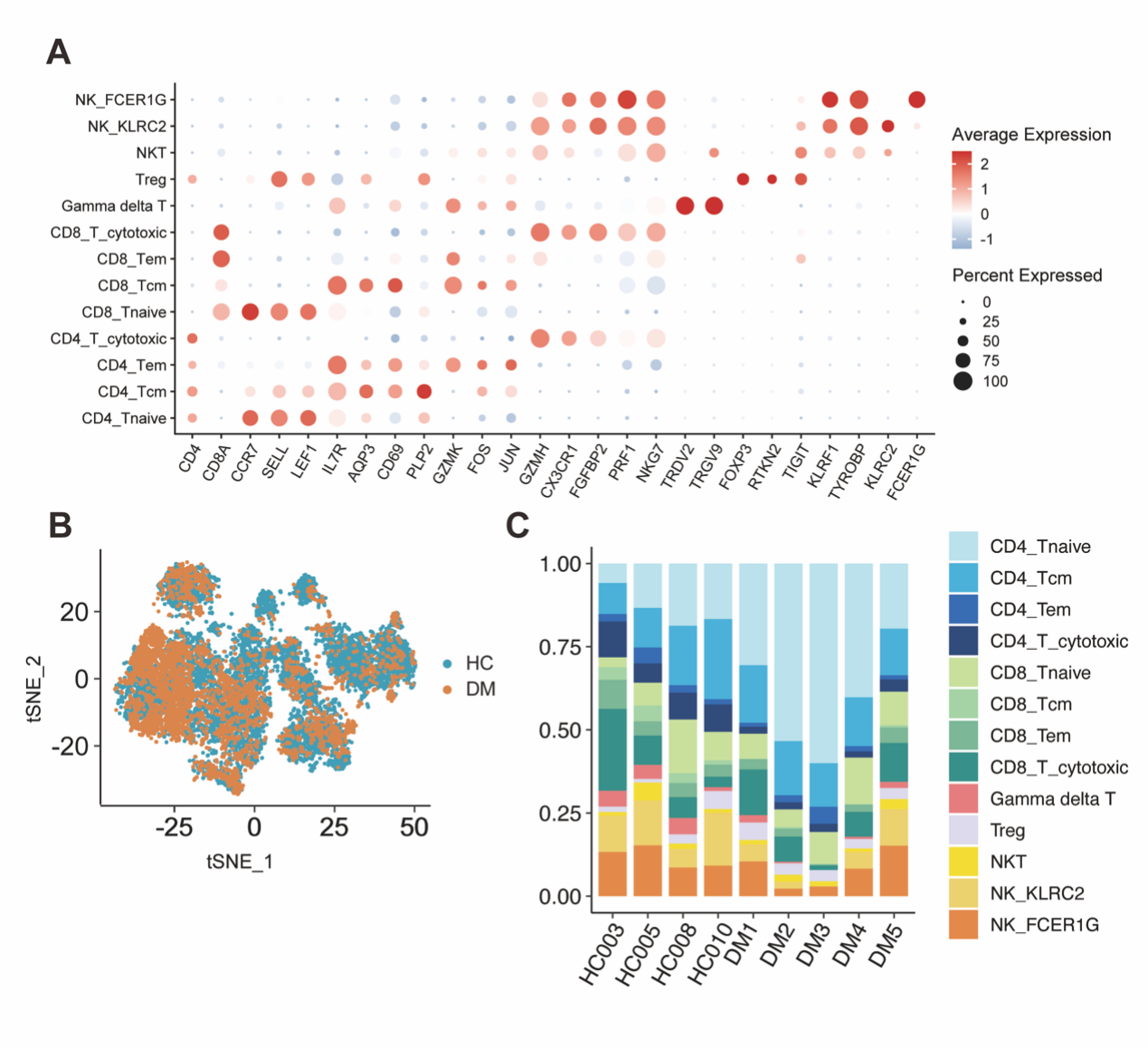


**Figure S5 Markers and proportions of T and NK cell compartments in PBMCs, related to Figure 5.**

**(A)** Dot plot illustrating hallmark gene expression across T and NK cell clusters in PBMCs.

**(B)** t-SNE plot showing T and NK cells across HCs and patients.

**(C)** Bar plot depicting the proportions of each cluster in each studied subject.


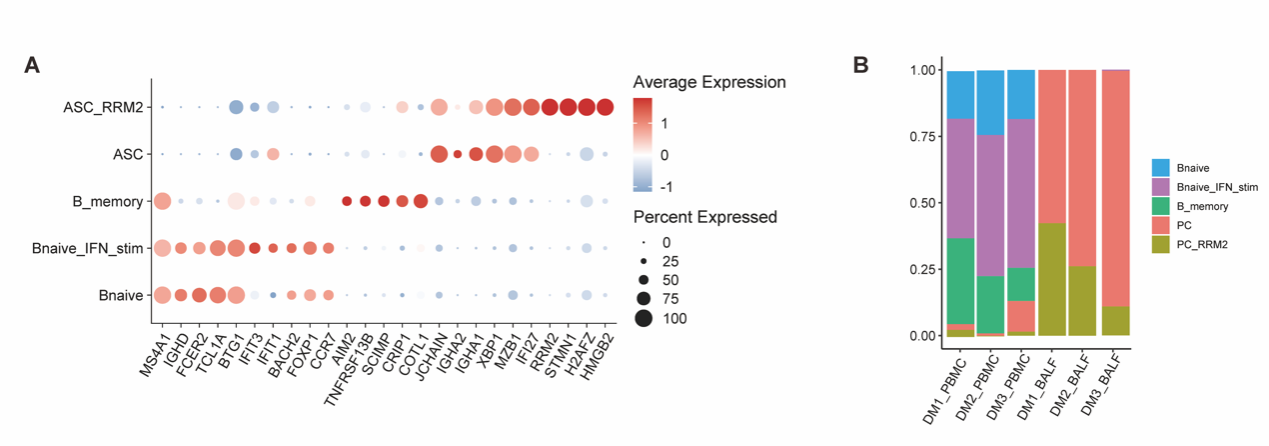


**Figure S6 Annotation and proportions of B cell compartments in PBMCs and BALF, related to Figure 6.**

1. Dot plot illustrating hallmark gene expression across B cell clusters.
2. Bar plot depicting the proportions of each B cell cluster in each studied subject.

**Table S1 Clinical characteristics of 5 anti-MDA5+ DM samples.**

| Characteristics | DM1 | DM2 | DM3 | DM4 | DM5 |
| --- | --- | --- | --- | --- | --- |
| Age, years | 57 | 53 | 52 | 55 | 55 |
| Sex | Female | Female | Male | Female | Female |
| MSA/MAA | Anti-MDA5-Ab+， anti-Ro52-Ab+++ | Anti-MDA5-Ab+,  anti-TIF1γ-Ab+,  anti-Ro52-Ab++ | Anti-MDA5-Ab++， anti-Ro52-Ab+ | Anti-MDA5-Ab+++, anti-Ro52-Ab+ | Anti-MDA5-Ab+++ |
| Smoking years | 0 | 0 | 40 | 0 | 0 |
| Disease duration, months | 6 | 6 | 2 | 8 | 3 |
| Fever | / | / | / | / | / |
| Productive cough | / | √ | √ | √ | / |
| Heliotrope rash | √ | √ | √ | √ | √ |
| Mechanic’s hands | / | / | √ | / | √ |
| Gottron’s papule/sign | √ | √ | √ | √ | √ |
| Muscle weakness | √ | / | √ | / | / |
| Arthritis/arthralgia | / | √ | √ | √ | √ |
| Dysphagia | / | / | / | / | / |
| Creatine Kinase, U/l | 16 | 46 | 80 | 30 | 66 |
| CRP, mg/L | 13.32 | 13.82 | 2.3 | 5.46 | 0.6 |
| ESR, mm/h | 62 | 108 | 27 | 53 | 12 |
| Ferritin, ng/ml | 1675 | 967 | 2522 | 166 | 349 |
| LDH, U/l | 463 | 320 | 380 | 497 | / |
| SpO2 | 100% with HFNC 20L/min | 100% with NC 3L/min | 95% with NC 3L/min | 93% at room air | 97% at room air |
| Treatment before BAL | MP, CysA | MP | MP, CTX, TAC | MP, TAC | MP |
| Treatment after BAL | MP, TAC, CysA, TCZ,  plasma exchange | MP, IVIG, TAC, TCZ | MP, TAC, CYC, TCZ, IVIG | MP, TAC, RUX | MP, CTX, TAC, APR |
| Prognosis | Dead | Alive | Dead | Alive | Alive |
| Death causes | Respiratory failure | / | Respiratory failure | / | / |

CRP: C-reactive protein; ESR: erythrocyte sedimentation rate; LDH: lactate dehydrogenase; NC: nasal cannula; HFNC: high flow nasal cannula; MP: methylprednisolone; CysA: cyclosporin A; CTX: cyclophosphamide; TAC: tacrolimus; IVIG: intravenous immunoglobulin; TCZ: tocilizumab; RUX: ruxolitinib; APR: apremilast

**Table S2 Demographic information of healthy controls.**

|  | PBMC (GSE158055) | | | | BALF (GSE145926) | | |
| --- | --- | --- | --- | --- | --- | --- | --- |
| Characteristics | HC003 | HC005 | HC008 | HC010 | HC1 | HC2 | HC3 |
| Age, years | 46 | 37 | 44 | 58 | 38 | 24 | 22 |
| Sex | Male | Female | Male | Male | Female | Male | Male |
| City | Chongqing | Shanghai | Beijing | Beijing | / | / | / |
| Chronic basic disease | / | / | / | / | None | None | None |
| Medication history | / | / | / | / | None | None | None |
| Samples | fresh PBMC | fresh PBMC | fresh PBMC | fresh PBMC | fresh BALF | fresh BALF | fresh BALF |

**Table S3 Clinical and laboratory data of anti-MDA5+ DM patients without immunosuppressive treatment (derived from published literature^3^)**

| Donor | Ages (years) | Gender | Disease Duration (mouth) | Ferritin (ug/L) | ATL (U/L) | ESR | LDH (U/L) | Ro-52 | ILD |
| --- | --- | --- | --- | --- | --- | --- | --- | --- | --- |
| MDA5_1 | 71 | F | 1 | 6198 | 259 | 30 | 428 | Positive | Yes |
| MDA5_2 | 30 | M | 2 | 468 | 104 | 7 | 264 | Negative | Yes |
| MDA5_3 | 50 | F | 2 | 160 | 75 | 25 | 337 | Negative | Yes |
| MDA5_4 | 58 | F | 2 | 593 | 61 | 16 | 411 | Positive | Yes |
| MDA5_5 | 62 | F | 3 | 2331 | 43 | 67 | 245 | Positive | Yes |

ALT: alanine aminotransferase; ESR: erythrocyte sedimentation rate; LDH: lactate dehydrogenase; ILD: interstitial lung disease

**Table S4 The reference genes to calculate scores.**

| Score | Genes |
| --- | --- |
| M1 | Cd86, Cd83, Dnase1l3, Cdh17, Ccl17, Cd74, RT1-Db1, Bcl2a1, Ccdc146, Fgl2, RT1-Da, Aspa, Cxcl16, Cyp11b3, Irf8, Cd63, Ctss, C1qa, Slamf7, Otulinl, Cldn1, Tagln2, Lynx1, AABR07069128.1, RT1-Ba, Klra22, Ms4a4c, Prr15, Mmp12, Hepacam2 |
| M2 | Mrc1, Pf4, Adgre1, Apoe, Lgmn, Dab2, Folr2, Glul, Ms4a7, Clec4a3, Cd163, Clec4a1, Tmem37, Ctsa, Ctsc, Plxna1, Cltc, Csf1r, Smpdl3a, Cndp1, Cfh, Scpep1, Slc43a2, Apobec1, Selenop, Ehd4, Lyve1, Ctsl, Snx5, Ncoa4 |
| Inflammatory | ABCA1, ABI1, ACVR1B, ACVR2A, ADGRE1, ADM, ADORA2B, ADRM1, AHR, APLNR, AQP9, ATP2A2, ATP2B1, ATP2C1, AXL, BDKRB1, BEST1, BST2, BTG2, C3AR1, C5AR1, CALCRL, CCL17, CCL2, CCL20, CCL22, CCL24, CCL5, CCL7, CCR7, CCRL2, CD14, CD40, CD48, CD55, CD69, CD70, CD82, CDKN1A, CHST2, CLEC5A, CMKLR1, CSF1, CSF3, CSF3R, CX3CL1, CXCL10, CXCL11, CXCL6, CXCL8, CXCL9, CXCR6, CYBB, DCBLD2, EBI3, EDN1, EIF2AK2, EMP3, EREG, F3, FFAR2, FPR1, FZD5, GABBR1, GCH1, GNA15, GNAI3, GP1BA, GPC3, GPR132, GPR183, HAS2, HBEGF, HIF1A, HPN, HRH1, ICAM1, ICAM4, ICOSLG, IFITM1, IFNAR1, IFNGR2, IL10, IL10RA, IL12B, IL15, IL15RA, IL18, IL18R1, IL18RAP, IL1A, IL1B, IL1R1, IL2RB, IL4R, IL6, IL7R, INHBA, IRAK2, IRF1, IRF7, ITGA5, ITGB3, ITGB8, KCNA3, KCNJ2, KCNMB2, KIF1B, KLF6, LAMP3, LCK, LCP2, LDLR, LIF, LPAR1, LTA, LY6E, LYN, MARCO, MEFV, MEP1A, MET, MMP14, MSR1, MXD1, MYC, NAMPT, NDP, NFKB1, NFKBIA, NLRP3, NMI, NMUR1, NOD2, NPFFR2, OLR1, OPRK1, OSM, OSMR, P2RX4, P2RX7, P2RY2, PCDH7, PDE4B, PDPN, PIK3R5, PLAUR, PROK2, PSEN1, PTAFR, PTGER2, PTGER4, PTGIR, PTPRE, PVR, RAF1, RASGRP1, RELA, RGS1, RGS16, RHOG, RIPK2, RNF144B, ROS1, RTP4, SCARF1, SCN1B, SELE, SELENOS, SELL, SEMA4D, SERPINE1, SGMS2, SLAMF1, SLC11A2, SLC1A2, SLC28A2, SLC31A1, SLC31A2, SLC4A4, SLC7A1, SLC7A2, SPHK1, SRI, STAB1, TACR1, TACR3, TAPBP, TIMP1, TLR1, TLR2, TLR3, TNFAIP6, TNFRSF1B, TNFRSF9, TNFSF10, TNFSF15, TNFSF9, TPBG, VIP |
| MHC class II | HLA-DMA, HLA-DMB, HLA-DPA1, HLA-DPB1, HLA-DQA1, HLA-DQB1, HLA-DRA, HLA-DRB1, HLA-DRB5 |
| Calprotectin protein | S100A1, S100A2, S100A3, S100A4, S100A5, S100A6, S100A7, S100A7A, S100A7L2, S100A7P1, S100A7P2, S100A8, S100A9, S100A10, S100A11, S100A12, S100A13, S100A14, S100A15A, S100A16, S100B, S100G, S100P, S100Z |
| Cytokine | IL2, IL7, CSF3, CXCL10, CCL2, CCL3, TNF, TFTN1, IL6, CCL7, IL1RN, CSF1, IFNG, IL2RA, IL10, IL18, HGF, CXCL9, CSF3, CCL27, IL1B, LTA, LTB, TNFSF13, IL18, IL4, CCL12, CXCL8, CXCL11, CCL4, CXCL1, CXCL2, CXCL3, CCL3L1, CCL8, CXCL16, IFNA1, IL1RN, CCL5, CCL11, HGF, IFNA2, CCL20, CCL3L1, CCL4L2, OSM, TNFSF14, SA100A12, FGF19, CXCL5, CCL19, IL18R1, TGFA, IFNB1, IL17C, TNFSF10, FGF7, XCL1, FGF13, LIF, TGFB3, INHBE, CERS1, TXLNA, IFNW1, IL22, XCL2, CCL25, CCL16, CD40LG, IL20, FASLG, TPO, SCYL3, PF4V1, TNFSF8, GDF15, IL1A, VEGFA, GDF7, BMP6, PDGFA, IL21, ABCD-1, ABCD-2, PDGFB, TNFSF4, FAM19A1, HBEGF, PDGFD, IL12RB2, GH1, VEGFB, MIP3B, IL27, PF4, BMP8B, TNFSF12, IL15, SCYL2, SCYL1, TSLP, GDF11, SDF1B, INHBA, PPBP, FGF11, IFNG-AS1, FGF22, VEGFC, CCL18, TNFSF11, IL12A, EBI3, AMH, IL26, IL32, PDGFC, FGF23, IGF1, IL1F11, CCL28, CLCF1, TNFSF9, BMP3, IL24, GDF10, CXCL6, GDF9, IL23A, IL16, CD70, IL5, FGF9, IFNL1, TSC1, FGF2, IL23R, IL1G, SPP1, IL12RB1, BMP4, IL13, TPAR1, TGFB2, FAM19A2, AGIF3, EDA, MIF, TNFSF13B, BMP7, FGF18, CCL23 |

**Supplemental References:**

1. Reyfman PA, Walter JM, Joshi N, et al. Single-Cell Transcriptomic Analysis of Human Lung Provides Insights into the Pathobiology of Pulmonary Fibrosis. *American journal of respiratory and critical care medicine*. Jun 15 2019;199(12):1517-1536. doi:10.1164/rccm.201712-2410OC

2. Morse C, Tabib T, Sembrat J, et al. Proliferating SPP1/MERTK-expressing macrophages in idiopathic pulmonary fibrosis. *The European respiratory journal*. Aug 2019;54(2)doi:10.1183/13993003.02441-2018

3. He J, Liu Z, Cao Y, et al. Single-cell landscape of peripheral immune response in patients with anti-melanoma differentiation-associated gene 5 dermatomyositis. *Rheumatology (Oxford, England)*. Nov 6 2023;doi:10.1093/rheumatology/kead597
